# Variations in Head and Neck Treatment Plan Quality Assessment Among Radiation Oncologists and Medical Physicists in a Single Radiotherapy Department

**DOI:** 10.3389/fonc.2021.706034

**Published:** 2021-10-12

**Authors:** Elisabetta Cagni, Andrea Botti, Linda Rossi, Cinzia Iotti, Mauro Iori, Salvatore Cozzi, Marco Galaverni, Ala Rosca, Roberto Sghedoni, Giorgia Timon, Emiliano Spezi, Ben Heijmen

**Affiliations:** ^1^ Medical Physics Unit, Azienda Unità Sanitaria Locale Istituto di Ricovero e Cura a Carattere Scientifico (USL-IRCCS) di Reggio Emilia, Reggio Emilia, Italy; ^2^ School of Engineering, Cardiff University, Cardiff, United Kingdom; ^3^ Department of Radiation Oncology, Erasmus MC Cancer Institute, Rotterdam, Netherlands; ^4^ Radiotherapy Unit, Azienda USL-IRCCS di Reggio Emilia, Reggio Emilia, Italy

**Keywords:** radiotherapy treatment planning, head and neck cancer, subjective plan quality assessment, inter-observer and intra-observer variation, automated treatment planning

## Abstract

**Background:**

Agreement between planners and treating radiation oncologists (ROs) on plan quality criteria is essential for consistent planning. Differences between ROs and planning medical physicists (MPs) in perceived quality of head and neck cancer plans were assessed.

**Materials and Methods:**

Five ROs and four MPs scored 65 plans for in total 15 patients. For each patient, the clinical (CLIN) plan and two or four alternative plans, generated with automated multi-criteria optimization (MCO), were included. There was always one MCO plan aiming at maximally adhering to clinical plan requirements, while the other MCO plans had a lower aimed quality. Scores were given as follows: 1–7 and 1–2, not acceptable; 3–5, acceptable if further planning would not resolve perceived weaknesses; and 6–7, straightway acceptable. One MP and one RO repeated plan scoring for intra-observer variation assessment.

**Results:**

For the 36 unique observer pairs, the median percentage of plans for which the two observers agreed on a plan score (100% = 65 plans) was 27.7% [6.2, 40.0]. In the repeat scoring, agreements between first and second scoring were 52.3% and 40.0%, respectively. With a binary division between unacceptable (scores 1 and 2) and acceptable (3–7) plans, the median inter-observer agreement percentage was 78.5% [63.1, 86.2], while intra-observer agreements were 96.9% and 86.2%. There were no differences in observed agreements between RO–RO, MP–MP, and RO–MP pairs. Agreements for the highest-quality, automatically generated MCO plans were higher than for the CLIN plans.

**Conclusions:**

Inter-observer differences in plan quality scores were substantial and could result in inconsistencies in generated treatment plans. Agreements among ROs were not better than between ROs and MPs, despite large differences in training and clinical role. High-quality automatically generated plans showed the best score agreements.

## 1 Introduction

Advanced radiotherapy delivery approaches such as intensity-modulated radiation therapy (IMRT) and volumetric modulated arc therapy (VMAT) have substantially increased opportunities for sparing organs at risk (OARs) with proven clinical impact (1–5). Ideally, for each individual patient, the applied treatment plan maximally exploits the full potential of the applied delivery technique. Currently, most treatment plans are generated with interactive trial-and-error planning (“manual planning”). It is well-known that plan quality in manual planning may be suboptimal, e.g., depending on experience and ambition of the planner, and on allotted planning time (6, 7). In recent years, several systems for automated plan generation have been developed, often resulting in enhanced plan quality compared with manual planning (8–13).

In both manual and automated planning, human evaluation and judgment of treatment plans are crucial. Normally, plans are produced by medical physicists (MPs) or dosimetrists and presented to treating radiation oncologists (ROs) for approval. During manual plan generation, planners usually develop a range of (intermediate) plans, but generally only a single plan or sometimes two competing plans are discussed with the RO. Prior to approval, the RO may request for adaptation of presented plans. A necessary assumption for this workflow to work well is that (unknown) disparity between planners and ROs on characteristics of good/optimal plans is absent or minor. In case of large disparity, a plan with high quality from the planner’s point of view may be presented to the RO, while a different plan with lower quality according to the planner, but clearly more attractive to the RO if she/he would have been aware of it, is intentionally not generated or presented. In such cases, there is no guarantee that plan modifications are requested and, if requested, to what extent the adapted plans would satisfy the needs of the RO.

In this study, we have systematically investigated differences between five ROs and four planning MPs, all working in a single radiotherapy department, in perceived quality of head and neck (HN) cancer plans. With the use of automated planning, multiple plans were generated per patient. Plan quality was scored using visual analogue scales.

## 2 Material and Methods

### 2.1 Patients and Clinical Treatment Plans

Planning CT data, contoured structures, and the clinical (CLIN) plan of 15 arbitrarily selected oropharyngeal HN cancer patients, recently treated with radiotherapy at Azienda USL-IRCCS Hospital (AUSL) of Reggio Emilia (Italy), were included in this study. Following American Joint Committee on Cancer (AJCC) TNM staging (14), six patients were classified as T2N2, three as T1N2, three as T2N1, and three as T4N2. Bilateral neck was irradiated in all patients. A simultaneous integrated boost (SIB) technique was used for all patients, delivering the prescribed doses in 33 daily fractions. Total doses for PTVhigh, PTVmedium, and PTVlow were 69.96, 59.4, and 54 Gy, respectively (15–17). For each planning target volume (PTV), the goal was to deliver 100% of the prescribed dose to 95% of the volume. All plans were normalized so that exactly 95% of PTVhigh received the prescription dose. Sizes of the involved PTVs were as follows: 178.5 ± 97.3 cm^3^ [63.3, 409.6], 208.4 ± 105.7 cm^3^ [39.8, 431.7], and 184.8 ± 51.0 cm^3^ [95.2, 248.7] for PTVhigh, PTVmedium, and PTVlow, respectively. OARs considered in planning were spinal cord, brainstem, left and right parotid, esophagus, oral cavity, larynx, mandible, pharyngeal constrictor muscles, and submandibular glands (17). Plans were generated using the following priorities for achieving planning objectives: 1) sparing of brainstem, optic chiasm, and spinal cord (so higher priority than PTV coverage); 2) achievement of PTV dose objectives in the order PTVhigh, PTVmedium, and PTVlow; 3) parotid gland sparing; and 4) sparing of other OARs and healthy tissues. The clinical planning protocol was largely in line with international protocols, such as RTOG (18–21) and JAVELIN protocols (22).

Patients were treated with 3-arc 6-MV VMAT delivered with a TrueBeam STx linac (Varian Medical Systems, Palo Alto, USA) (10 patients) or using TomoTherapy (Accuray Inc., Sunnyvale, USA) (five patients). Clinical planning was performed with the Eclipse Treatment Planning System (TPS) v. 13 (Varian Medical Systems, Palo Alto, USA) or Tomoplan v. 3-4 (Accuray Inc., Sunnyvale, USA).

### 2.2 Global Study Design

Apart from the CLIN plan, two (for five patients) or four (for 10 patients) additional VMAT plans were evaluated in this study, resulting in a total of 65 evaluable plans. The extra plans had variable plan quality and were generated with automated planning (details in Section 2.5). Each of the 65 available plans was evaluated by five departmental ROs (three with more than 5 years of experience in HN radiotherapy and two with less than 1 year of experience) and four MPs (all with more than 5 years of experience), resulting in a total of 585 subjective plan evaluations. These involved ROs and MPs represented all involved staff in HN treatment in our department at the time of the study.

For each patient, every observer independently gave a score to each of the 3 or 5 available plans in a single session (details in Section 2.3). Scoring was blinded; i.e., observers did not know how the plans were generated. Apart from giving a quality score to each plan, observers were also asked what change they considered most desirable for improvement of the plan (without knowing whether this would be feasible or not); see also Section 2.3.

To assess intra-observer variability in quality scoring, one RO and one MP performed the entire scoring process for 65 plans a second time, with a delay of at least a month. Previous results were blinded.

### 2.3 Plan Scoring Procedure

For each patient, all available dose distributions were simultaneously imported into the Eclipse TPS and linked to a virtual plan without any mention of the original delivery approach (VMAT or TomoTherapy), plan geometry, machine parameters, etc. With all plans simultaneously open, the observer gave a separate 1–7 score to each plan, following the routine procedure for plan evaluation (inspection of 3D dose distribution, dose–volume histogram (DVH) data, etc.), with higher scores pointing at perceived higher quality: 1–2, unacceptable (plan category 1); 3–5, acceptable if further planning would not have resulted in a better plan (this planning was not performed in this study) (plan category 2); and 6–7, acceptable, no further planning needed (plan category 3). A 7-point scale was chosen because of good performance in psychometric literature (23–25). In the remainder of this paper, the 1–7 scores are denoted “raw” scores, while plan categories 1–3 define the more intuitive “category” scores. The applied division of the raw scores in categories was made before the start of subjective plan scoring. As is visible in **Supplementary Figure S1**, this division was also explicitly shown to the observers while giving scores to plans. For the analyses, another scoring system was introduced as well, the so-called “binary” scoring system: raw scores 1 and 2 were grouped as binary score 0 (plan is unacceptable), and raw scores 3–7 were given binary score 1 (plan is in principle acceptable).

To express the most urgent need for plan improvement, the observers could choose from A) PTVs (coverage, conformity, and homogeneity), B) OAR group 1 (spinal cord, brainstem, and optical system), C) OAR group 2 (parotids, mandible, oral cavity, larynx, and esophagus), D) unspecified normal tissue, or E) none. See also **Supplementary Figure S1**.

### 2.4 Evaluation of Inter-Observer Differences in Plan Scoring

With nine observers, there were in total 36 unique combinations of two observers, here designated as “pairs”. To analyze inter-observer differences in perceived plan quality, for all these observer pairs, percentages of agreement and disagreement in the scores given to the 65 evaluated plans were established. Analyses were partially based on raw scores, category scores, and binary scores. Observed percentages of agreement in RO–RO pairs and MP–MP pairs were compared with percentages of agreement in RO–MP pairs. Suggested most desired plan improvements were used to generate for each observer separately a frequency analysis of provided suggestions for the 65 evaluated plans.

### 2.5 Automatically Generated MCOa and MCOx Plans

Autoplans were generated with the Erasmus-iCycle system for fully automated multi-criteria optimization (MCO) (10, 26). Plan optimization in Erasmus-iCycle is based on so-called wish-lists (WLs), containing hard planning constraints and planning objectives with goal values and assigned priorities. A dedicated WL is needed for every treatment site. In essence, the WL defines an optimization protocol for automated multi-criteria generation of a single Pareto-optimal treatment plan for each patient. The aim in WL creation is to maximally ensure the highest clinical quality of the generated Pareto-optimal plans, in line with the clinical planning protocol and tradition [Appendix 10]. Also, in this study, such a WL was created with input of all ROs and MPs involved in the study (WLa). In the remainder of the paper, plans generated with WLa are denoted as “MCOa.” These MCOa plans consisted of 23 equi-angular IMRT beams, with high similarity to VMAT and avoiding time for segmentation (27–29). With WLa as a starting point, 20 alternative WLs, “WLx” (x = b, c, d, …), were created for generation of “MCOx” plans. The WLx were derived from WLa by randomly varying the priorities of PTVmedium and PTVlow objectives and of the OARs. For generation of an MCOx plan for a patient, one of the 20 WLx was randomly selected; and in addition, the number of beams was randomly varied between 10 and 23. As for WLa, the 20 WLx enforced adherence to the hard planning constraints for brainstem, optic chiasm, and spinal cord, as in clinical planning (above). At the same time, the WLx allowed generation of MCOx plans with a spread in dosimetric differences compared with the corresponding MCOa plans. For patients 1–10, the CLIN plan was supplemented with the MCOa plan and three MCOx plans (in total five evaluable plans). For patients 11–15, apart from the CLIN and MCOa plan, there was one additional MCOx plan used in this study (three evaluable plans in total). The switch from five to three plans is discussed in Section 4. For putting the subjective scoring of plan quality by observers in context, dosimetric characteristics of CLIN, MCOa, and MCOx plans were analyzed by mutual comparisons of dosimetric plan parameters and DVHs.

### 2.6 Statistical Analysis

Shapiro’s test and Student’s t-test were used to assess the normality of distributions and statistical significance of dosimetric differences between plans generated with different planning approaches, i.e., CLIN, MCOa, and MCOx. Wilcoxon’s two-sided signed-rank tests were used to assess statistical significance of mean score differences between CLIN, MCOa, and MCOx. Differences were considered significant if p < 0.05.

To assess statistical significance (0.05 level) of observed percentages of agreement for the 65 plan scores of the two observers in an observer pair, binomial distributions were used to calculate probabilities of percentage agreements in case of complete uncorrelated (random) choices of the two observers in a pair. To this end, success probabilities p of 1/7, 1/3, and 1/2 were used for raw, category, and binary scores, respectively.

The percentages of agreement in plan scores between the two observers in observer pairs were also analyzed with Cohen’s coefficient (K) (30). The relative strength of agreement between the two observers in a pair is dependent on the calculated K-value. Landis and Koch (31) have proposed the following classification: K < 0, agreement “poor”; 0 ≤ K ≤ 0.2, agreement “slight”; 0.2 < K ≤ 0.4, agreement “fair”; 0.4 < K ≤ 0.6, agreement “moderate”; 0.6 < K ≤ 0.8, agreement “substantial”; and 0.8 < K ≤ 1, agreement “almost perfect”. For binary scoring, the resulting number of samples for unapproved status was not enough to achieve significant confidence limits in Cohen’s coefficients for many evaluators (32). Therefore, Cohen’s analyses were only performed for raw and category scores.

One-way ANOVA tests were performed to assess statistical significance of differences in percentages of agreement between subgroups of observers, 1) only RO–RO, 2) only MP–MP, and 3) only RO–MP pairs, after having assessed the normality of the distribution with the Kolmogorov–Smirnov test. Bartlett’s test was used to test the homogeneity of variance. When ANOVA assumptions were not met, the Kruskal–Wallis rank sum test was used as non-parametric alternative to one-way ANOVA. The Wilcoxon’s signed-rank test was used to test agreement differences between CLIN and MCOa plans, expert and no-expert ROs, and three and five evaluated plans per patient.

## 3 Results

### 3.1 Differences Between Evaluated Clinical, MCOa, and MCOx Plans in Dosimetry

In panels a) and c) of **Supplementary Figure S2**, median DVHs for the CLIN, MCOa, and MCOx plans are presented, showing for each dose the corresponding median volume in the considered plans. For individual patients, the DVH differences between the CLIN, MCOa, and MCOx plans were pairwise quantified by generating differential DVHs: volume differences as a function of dose. Median volume differences and 10th and 90th percentiles are presented in panels b) and d) of **Supplementary Figure S2**. The 10th and 90th percentile curves point at large inter-patient variations in DVH differences between CLIN, MCOa, and MCOx plans. **Supplementary Table S1** shows how the DVH differences translate in differences in dosimetric plan parameters. Only few of the differences between CLIN, MCOa, and MCOx plan parameters were statistically different, while ranges were very broad. This is in line with the observations in **Supplementary Figure S2**. **Supplementary Figure S3** presents for each of the 15 study patients separately an overview of the dosimetric differences between the included three to five treatment plans.

### 3.2 Scoring for an Example Patient

**Figure 1:**
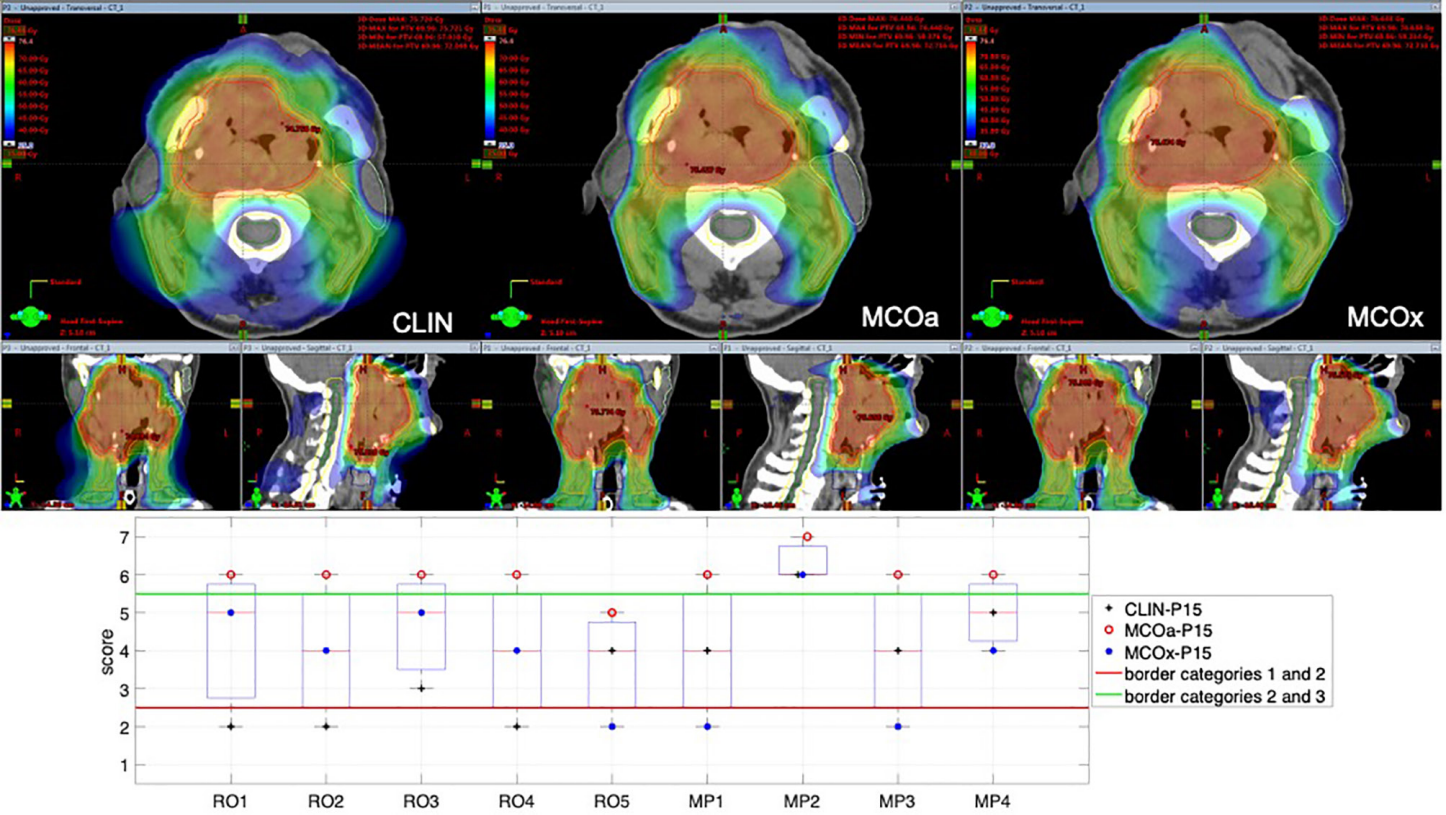
Differences in subjective plan scores among the nine observers in the study, illustrated with an example patient (patient 15). Top panels: dose distributions of the evaluated CLIN, MCOa, and MCOx plans in axial, sagittal, and frontal planes. Bottom panel: corresponding subjective plan quality scores for the CLIN, MCOa, and MCOx plans of patient 15 for each of the nine observers, five ROs (RO1–RO5), and four MPs (MP1–MP4). Plans below the horizontal red line are considered unacceptable (raw scores 1 and 2, category 1). Above the green line are plans that can straightaway be delivered without any attempt to further improve the plan (raw scores 6 and 7, category 3). In the middle are the plans that are acceptable if further planning would not result in significant improvements (raw scores 3–5, category 2). CLIN, clinical; RO, radiation oncologist; MP, medical physicist.

To introduce the type of scoring data obtained for each patient, **Figure 1** shows the raw scores of the nine observers for the CLIN, MCOa, and MCOx plans of study patient 15, a patient showing large scoring variations. The majority of observers (6/9) selected MCOa as the best plan, while MCOx was selected most as the worst plan (5/9). This ranking of MCOa and MCOx is in line with the applied WLs for generation of these plans (Section 2.5). However, for all three plans, there were large inter-observer differences in raw scores (2–5 for MCOx and 2–6 for CLIN and MCOa). RO4 scored the clinically delivered CLIN plan as unacceptable, while for MP1, this plan was acceptable without further planning attempts. For RO3, MCOa was unacceptable, while for MP2, it could be delivered straightaway. **Figure 1** also shows large inter-observer differences in score ranges. As demonstrated in the group analyses below, large scoring variations were observed for all patients and the vast majority of plans.

### 3.3 Radiation Oncologist Experience in Head and Neck Radiotherapy and Scoring

As mentioned in Section 2.2, three participating ROs had more than 5-year experience in HN radiotherapy, while the other two had less than 1-year experience for this tumor site. When considering the raw, category, and binary scores of all 65 plans, median values for all five ROs/only three expert ROs were 28.5%/36.9% (p = 0.5), 56.2%/61.6% (p = 1.0), and 75.4%/75.4% (p = 0.7), respectively. Based on these observations, it was decided that in further group analyses, the five ROs in this study were considered as a single group.

### 3.4 Differences Between Clinical, MCOa, and MCOx Plans in Observer Scores

**Table 1** reports differences between CLIN, MCOa, and MCOx in subjective scores, complimentary to the dosimetric differences in **Supplementary Table S1**. The automatically generated MCOa plans outperformed the clinically delivered CLIN plans, but for the binary scores, this was not statistically significant. Score differences were overall the largest between MCOa and MCOx and with the smallest p-values, with the former showing the highest scores, as to be expected from the respective WLs used for automated plan generation (Section 2.5).

**Table 1:** Median differences in raw, category, and binary scores assigned by the five ROs, four MPs, and all nine observers combined (All).

*Raw* scores	MCOa − CLIN				MCOx − CLIN				MCOx − MCOa			
	Diff	Min	Max	p	Diff	Min	Max	p	Diff	Min	Max	p
*All*	0.9	−1.4	2.8	**0.01**	−0.6	−2.9	2.8	**0.05**	−1.5	−1.2	3.2	**<0.001**
*ROs*	0.9	−1.4	3.2	**0.02**	−0.5	−2.7	3.2	0.15	−1.3	−1.4	3.2	**0.001**
*MPs*	0.9	−1.5	3.5	**0.02**	−0.8	−3.1	2.3	0.06	−1.6	−1.0	3.3	**<0.001**
***Category* scores**	**Diff**	**Min**	**Max**	**p**	**Diff**	**Min**	**Max**	**p**	**Diff**	**Min**	**Max**	**p**
*All*	0.3	−0.6	1.1	**0.02**	−0.3	−1.3	1.2	**<0.001**	−0.6	−0.6	1.4	**<0.001**
*ROs*	0.3	−0.4	1.4	**0.02**	−0.2	−1.0	1.4	**0.01**	−0.5	−0.6	1.4	**<0.001**
*MPs*	0.3	−1.0	1.5	0.1	−0.4	−1.8	1.0	<**0.001**	−0.7	−0.5	1.8	**<0.001**
***Binary* scores**	**Diff**	**Min**	**Max**	**p**	**Diff**	**Min**	**Max**	**p**	**Diff**	**Min**	**Max**	**p**
*All*	0.1	−0.1	0.4	0.1	−0.2	−0.9	0.4	**0.004**	−0.2	−0.1	0.8	<**0.001**
*ROs*	0.1	−0.2	0.6	0.2	−0.2	−1.0	0.6	**0.004**	−0.3	−0.2	0.8	**<0.001**
*MPs*	0.1	−0.3	0.5	0.1	−0.1	−0.8	0.5	**0.01**	−0.2	0.0	0.8	**<0.001**

Significant p-values are reported in bold.

RO, radiation oncologist; MP, medical physicist.

### 3.5 Inter-Observer Variability in Plan Quality Scores

**Figure 2:**
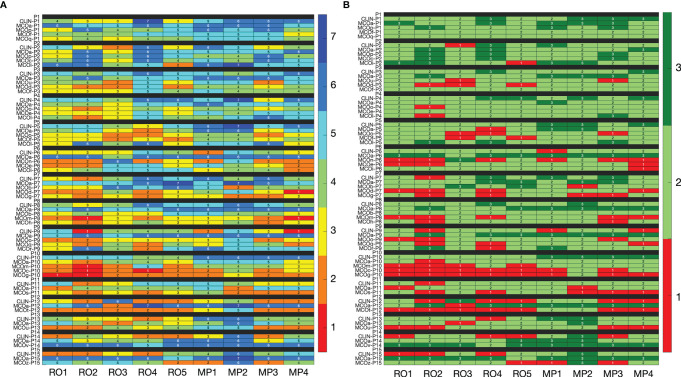
**(A)** Heatmap visualization for raw plan quality scores (1–7, with 7 indicating the highest quality) of the nine observers (x-axis) for all 65 included plans (y-axis). **(B)** Heatmap visualization for category scores derived from the raw scores. In panel B, the color red indicates that the plan is considered unacceptable (category 1, raw scores 1 and 2), while light and dark green (category 2 with raw scores 3–5, and category 3 with raw scores 6 and 7) indicate that the plan is in principle acceptable. In the binary scoring system, red has binary score 0, while both light and dark green have binary score 1. See Section 3.5 for interpretation.

In line with the observations for patient 15 (above), for the majority of plans, inter-observer variations in assigned scores were large (**Figure 2**). For the 65 evaluated plans, the average standard deviation (SD) for the nine raw observer scores was 1.06 [0.33, 1.56] (**Figure 2A**). For 29 of the 65 plans, all category scores (1, 2, and 3) were present in the nine scores (**Figure 2B**). For 15/65 plans, there was at least one observer that scored category 3 (acceptable without further planning attempts), while at the same time), there were also observers that considered the plan unacceptable (category 1). Considering all 65 plans, the median percentage of plans declared unacceptable by an observer was 18.8% ± 8.6% [6.2%, 35.4%]. For CLIN, MCOa, and MCOx plans separately, these percentages were 14.8% ± 9.9% [0.0, 33.3], 4.4% ± 4.7% [0.0, 13.3], and 26.7% ± 12.3% [8.6, 48.6], respectively. Kruskal–Wallis rank tests resulted in a statistically significant difference, with p = 0.005. Wilcoxon’s signed-rank test showed a statistically significant difference between MCOa and MCOx (p = 0.005), while for CLIN *vs*. MCOa, p = 0.1, and for CLIN *vs*. MCOx, p = 0.2.

**Figure 3:**
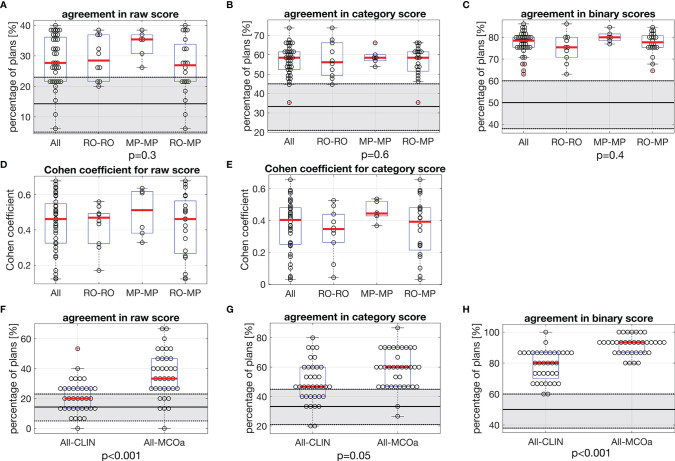
In each panel, horizontal red lines in the boxplots show median values, while the edges of the boxes are the 25th and 75th percentiles. The whiskers extend to the most extreme data points not considered outliers, and the outliers are plotted individually using the “+” symbol. **(A–C)** Each marker shows for one of the unique 36 observer pairs in this study the percentage of 65 evaluated plans for which they agree in **(A)** raw score, **(B)** category score, and **(C)** binary score. In each panel, the first boxplot includes the data for all 36 observer pairs (All). For the other three boxplots, the data are split according to subgroups of observer pairs: RO–RO, pairs consist of two ROs; MP–MP, pairs consist of two MPs; RO–MP, pairs consist of one RO and one MP. Gray zones show expected distributions of agreement percentages in case of random, uncorrelated scoring by the observers in a pair, with the expected value denoted by the solid black line and the 95% confidence interval shown by the dotted borders. For observed agreements outside the gray zones, the difference with the expected score for random scoring is statistically significant. The p-values relate to ANOVA tests between subgroups RO–RO, MP–MP, and RO–MP of all observer pairs. **(D, E)** Corresponding Cohen**’**s coefficients for raw and category scores. **(F–H)** Comparisons between observed agreement percentages for CLIN plans for all observer pairs, compared with observed agreement percentages for MCOa plans (generated with the optimal wish-list) for all observer pairs. The p-values were established with Wilcoxon**’**s signed-rank tests. Gray zones: as in panels **(A–C)**. RO, radiation oncologist; MP, medical physicist; CLIN, clinical.

**Figures 3A–C** show unique pairs of two observers, the percentages of plans for which they agreed in a plan score. Considering all 36 unique observer pairs in this study, the median percentage of agreement in raw plan scores was 27.7% [6.2, 40.0] (“all” boxplot in **Figure 3A**). In case of complete randomness in the scoring of two observers in a pair, an agreement percentage of 14.3% would be expected (horizontal solid line in gray zone). For category (**Figure 3B**) and binary scores (**Figure 3C**), these median percentages were 58.5% [35.4, 73.8] (33.3% expected in case of randomness) and 78.5% [63.1, 86.2] (50% in case of randomness), respectively. The vast majority of percentages of agreement in **Figures 3A–C** are outside the gray zones, meaning that they are statistically significantly different from the corresponding expected values for random scoring, indicated by the horizontal solid lines. With one-way ANOVA p-values of 0.3, 0.6, and 0.4, there were no differences between the observer pair subgroups RO–RO, MP–MP, and RO–MP in the agreement distributions in **Figures 3A–C**, respectively.

Cohen’s coefficient analyses for raw scores (**Figure 3D**) resulted in median K-values [range] of 0.46 [0.12, 0.68] when considering all observer pairs, 0.47 [0.17, 0.56] for ROs, 0.51 [0.33, 0.64] for MPs, and 0.46 [0.12, 0.68] for RO–MP. Following the labelling by Landis and Koch (M&M), the overall agreement is “moderate.” More in detail, considering all 36 observer pairs, 11% (N = 4) resulted in slight agreement, 25% (N = 9) in fair agreement, 47% (N = 17) in moderate agreement, and 17% (N = 6) in substantial agreement. For category score analyses (**Figure 3E**), Cohen’s median K-values [range] were 0.40 [0.03, 0.66] for all, 0.35 [0.04, 0.53] for ROs, 0.44 [0.37, 0.54] for MPs, and 0.39 [0.03-0.66] for RO–MP pairs. The overall agreement, in Landis and Koch scale, resulted in “fair”; 19% (N = 7) resulted in slight agreement, 31% (N = 11) in fair agreement, 47% (N = 17) in moderate agreement, and 3% (N = 1) in substantial agreement.

**Figures 3F–H** present scoring agreements for CLIN and MCOa plans separately, showing substantially better agreements for the automatically generated MCOa: when considering all 36 observer pairs, agreement percentages for CLIN/MCOa were 20.0%/33.3% (p < 0.001), 46.7%/60.0% (p = 0.005), and 80.0%/93.3% (p < 0.001) for raw, category, and binary scores, respectively.

### 3.6 Intra-Observer Variation in Plan Quality Scores

For the RO and MP involved in the intra-observer analyses, agreement percentages for the 65 initial raw plan scores and the 65 repeat raw scores were 40.0%/52.3% for RO/MP (N = 65). This is substantially higher than the expected percentage for random scoring (14.3%) and the median percentage of inter-observer score agreement of 27.7%; see **Figure 3A**. The repeat category agreements for the RO/MP were 70.8%/89.2% (N = 65) with corresponding expected random agreements and median inter-observer agreements of 33.3% and 58.5% (**Figure 3B**), respectively. For binary scoring, the RO/MP agreements were 86.2%/96.2%, with expected random and median inter-observer agreements of 50% and 78.5% (**Figure 3C**), respectively.

### 3.7 Suggested Plan Improvements

**Figure 4:**
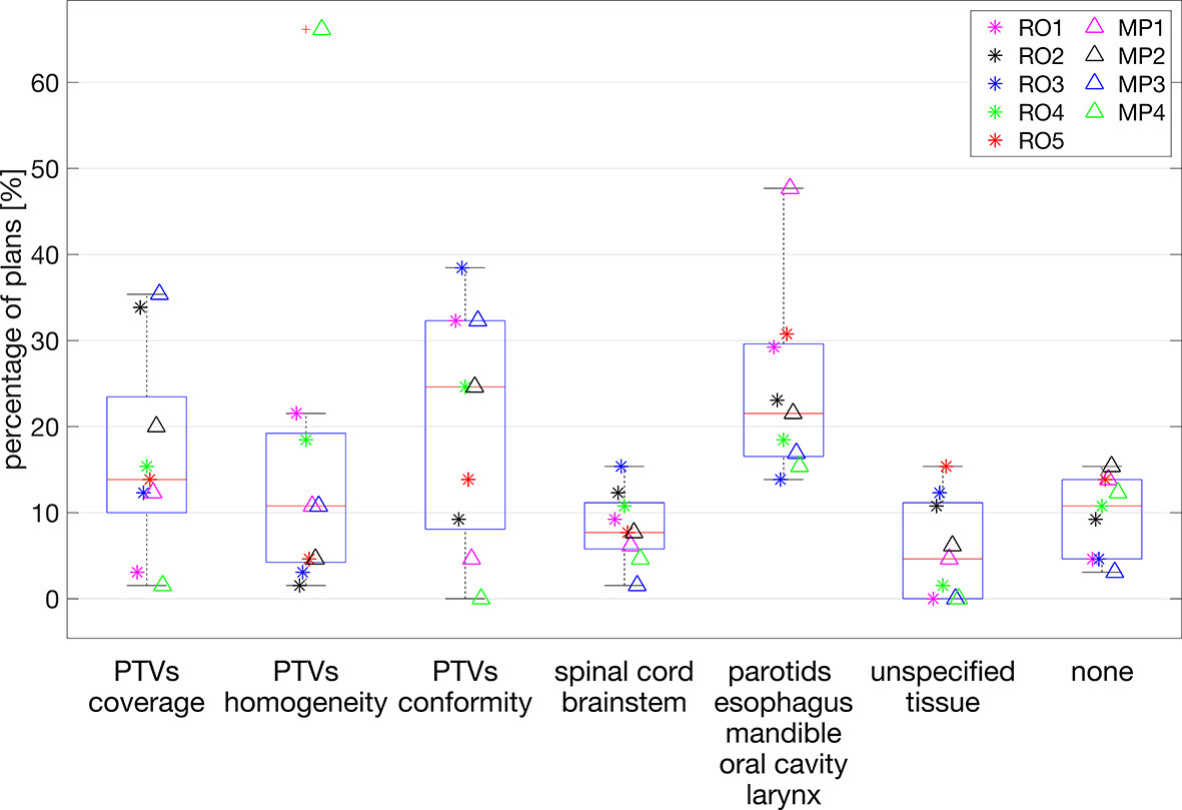
Percentages of plans (y-axis) for which plan improvement options along the x-axis were requested. Each marker indicates a selected observer the percentage of plans for which the corresponding option for plan improvement was selected. For each observer, the presented percentages add up to 100%.

Large variability between observers was also observed in the suggestions for plan improvement. **Figure 4** shows the variability between observers for each of the possible options for improvements. Overall, the most chosen options were PTV conformity and dose reductions in OAR of group 2 (**Supplementary Figure S1**), parotids, esophagus, mandible, oral cavity, and larynx, with median percentages of 24.6% [0.0, 38.5] and 21.5% [13.8, 47.7], respectively. In the intra-observer evaluations, the participating RO and MP showed agreement percentages in the request for plan improvement of 28% and 46%, respectively.

## 4 Discussion

In most centers, treatment plans are made by radiation therapy technologists (RTTs), dosimetrists, or MPs and evaluated for final approval by the treating ROs. The process, often denoted as manual planning or trial-and-error planning, may have several iterations in which the planner adjusts in-between plans, based on feedback by the RO. Limited common understanding or agreement between planners and ROs on how good plans should look like can result in suboptimal dose distributions, even with iteration loops. In this study, we have systematically investigated differences between five ROs and four planning MPs of a single radiotherapy department in perceived quality of oropharynx cancer plans. To the best of our knowledge, this is the first study that systematically investigates variations in subjective plan quality assessment among ROs and MPs working in a single department.

Even in our relatively small center with ROs and MPs working closely together based on the center’s planning protocol (which is in line with international protocols, see M&M), large variations in subjective plan scores were observed. Considering all 36 unique observer pairs, the median percentage of plans for which they disagreed on clinical acceptability was 21.5% (**Figure 3C**), with minimum/maximum disagreements between pairs of 13.8%/36.9%. Based on Landis and Koch’s labelling of Cohen’s kappa values, the overall agreements in raw and category scores were “moderate” and “fair,” respectively, but large variations between observer pairs were observed, going from “slight agreement” to “substantial agreement.”

As shown in **Supplementary Figures S2B, S2D, S3 **and** Supplementary Table S1**, dosimetric differences between the CLIN, MCOa, and MCOx plans could be substantial. As demonstrated in **Figure 2A**, for many observer–patient combinations, these dosimetric variations resulted in large variations in the three or five plan scores. On the other hand, different observers did often substantially disagree on the score of the same patient plan (see rows in **Figure 2A**). As can be observed in **Supplementary Figure S3**, dosimetric differences between patient plans, both positive and negative, were mostly not restricted to one parameter or one structure. Probably, different observers often appreciated the mixes in dosimetric pluses and minuses rather differently, contributing to the large disagreements between observers in assigned scores. This would be in line with the large inter-observer variations in suggested plan improvements (Section 3.7).

**Figures 3A–C** show that agreement percentages for RO–RO, MP–MP, and RO–MP pairs were similar (no statistically significant differences). This implicates that despite large differences in training and clinical roles of ROs and MPs, there were no enhanced rates of score mismatches in RO–MP pairs compared with RO–RO pairs.

Possibly, renewed, broad departmental discussions on plan requirements, aiming at a broadly shared and precisely defined view on plan quality, could improve the current large inter-observer variation in plan quality assessments. Probably also automated planning could result in improvements: as visible in **Figures 3F–H**, scoring agreements were better for the MCOa plans than for the CLIN plans, possibly related to more consistent automated generation of the MCOa plans. Apart from the better agreement between observers, MCOa scores were overall also higher than CLIN scores (**Table 1**), and MCOa plans were less frequently considered unacceptable than CLIN plans (4.4% *vs*. 14.8%, p = 0.1, Section 3.5). Enhanced plan quality with automated planning compared with manual planning has been observed previously [see, e.g., (8–12)], but to our knowledge, this is the first study showing also reduced inter-observer variations in subjective plan scores for the autoplans compared with corresponding manual plans. Other studies have pointed at the use of numerical plan quality assessment tools to enhance treatment plan quality (33).

In this study, clinical information about the patients was not available when doing the plan assessments, while it was available when the CLIN plan was made. This could in some cases have influenced scoring of the CLIN plan. On the other hand, all CLIN plans obeyed all hard clinical constraints for targets and OARs.

For some study patients, the CLIN plan was generated for TomoTherapy delivery, while the competitive MCOa and MCOx plans simulated VMAT (*Materials and Methods* section). Although observers were not informed on the delivery mode of presented plans, and all observers were aware that plan quality assessment was the study topic, it cannot be excluded that an observer could have identified TomoTherapy plans, which could possibly have influenced the scoring.

Although the observers were asked to give an absolute score (1–7) to each plan, the scoring of all three or five plans of a patient in a single session could have influenced the scores for the individual plans. For example, a plan could be perceived as unacceptable in the presence of a very good alternative plan, while when scored separately, the former plan could possibly have been acceptable for the observer. Such a mechanism could maybe in part explain the observation that 14.8% (median percentage for the nine observers, Section 3.5) of the CLIN plans was scored unacceptable, while all CLIN plans fulfilled the clinical hard constraints on PTV coverage, spinal cord Dmax, etc. It could maybe also explain the large difference between MCOa and MCOx in unacceptability rate (4.4% *vs*. 27.7% p = 0.005, Section 3.5), while also the intentionally suboptimal MCOx plans were generated while obeying all hard constraints (PTV, spinal cord, etc.). These observations point at a weak point of current manual planning: evaluating a plan is extremely difficult if there are no alternative plans.

In this study, we started off with five evaluable treatment plans per patient for the first 10 patients and then switched to three plans per patient to reduce the workload for the observers to a more acceptable level. For raw scores, the median of the percentage of score agreements for the 36 unique observer pairs was the highest for three plans per patient (33.3% *vs*. 26.0%), which was borderline significant (p = 0.07). For category scores, three plans resulted in lower agreement percentages (53.3% *vs*. 60%, p = 0.06), which was also seen for binary scores (66.7% *vs*. 81.0%, p < 0.001). Probably, the involved patient numbers are too small to draw strong conclusions on three versus five.

The 70.8% and 89.2% agreements in repeated category scoring and 86.2% and 96.2% in repeated binary scoring (Section 3.6) point at an option for high-accuracy score prediction for single observers with machine learning. Future application of such tools could possibly contribute to enhanced plan quality consistency. This is a topic of ongoing research.

In this study, we considered oropharynx cases with three dose levels and many OARs. The complexity of these cases could have contributed to the observed large and frequent disparities in observer scores. Possibly, for less complex tumor sites, agreement in plan scores could be better, which is a topic for further research.

We believe that this is the first study that quantitatively evaluates variations in subjective assessments of the same treatment plans by various observers (ROs and MPs) in the same department. Our study is very different from, but complementary to, other studies that demonstrate that different planners can generate very different plans for the same patient, even with very detailed, quantitative instructions on how the plan should look like (6). In the latter studies, plan quality differences are usually attributed to differences between planners in planning skills, dedication, and ambition, and in time spent on planning. On the contrary, in our study, all observers evaluate the same plans, and we test how well these plans fit the observer-specific ideas on how good plans should look like.

The results of the current study could stimulate similar studies in other departments as they seem to point at an important weak link in radiotherapy planning. It is commonly recognized that variations between ROs in delineated targets are a major concern in clinical radiotherapy. This study suggests that large inter-observer variations in plan quality assessments (even in a single department) could be another Achilles heel for successful treatment.

## 5 Conclusions

Inter-observer differences in treatment plan quality assessments in radiotherapy can be substantial and could hamper consistent preparation of high-quality plans, even in a single radiotherapy department. Agreements between ROs and MPs in plan assessments were similar to agreements among ROs only, despite large differences between ROs and MPs in training and clinical roles. Automatically generated plans (MCOa) showed the highest median scores and best inter-observer score agreements, pointing at a potential for automated planning to improve clinical practice.

## Data Availability Statement

The original contributions presented in the study are included in the article/supplementary material. Further inquiries can be directed to the corresponding author.

## Ethics Statement

The studies involving human participants were reviewed and approved by Comitato Etico di Area Vasta Emilia Nord—n. 391/2018/OSS/IRCCSRE. The patients/participants provided their written informed consent to participate in this study.

## Author Contributions

EC, BH, LR, AB, and ES contributed to the development of the study design and applied methodology. LR, EC, and AB developed the wish-lists for automated planning, in collaboration with CI, RS, MI, AR, GT, SC, and MG. CI, RS, MI, AR, GT, SC, MG, AB, and EC performed the subjective plan evaluations. EC, AB, LR, and BH performed the data analysis, with AB responsible for the statistical analyses. BH and EC supervised all work. All authors contributed to the article and approved the submitted version.

## Conflict of Interest

The authors declare that the research was conducted in the absence of any commercial or financial relationships that could be construed as a potential conflict of interest.

## Publisher’s Note

All claims expressed in this article are solely those of the authors and do not necessarily represent those of their affiliated organizations, or those of the publisher, the editors and the reviewers. Any product that may be evaluated in this article, or claim that may be made by its manufacturer, is not guaranteed or endorsed by the publisher.
