# Supplementary material for: Variations in Head and Neck Treatment Plan Quality Assessment Among Radiation Oncologists and Medical Physicists in a Single Radiotherapy Department
Source: Front Oncol. 2021 Oct 12;11:706034. doi: 10.3389/fonc.2021.706034 (PMC8545894; doi:10.3389/fonc.2021.706034)
Supplement: Supplementary file 1 [file Presentation_1.pdf]

Supplementary Material

1 Supplementary Figures

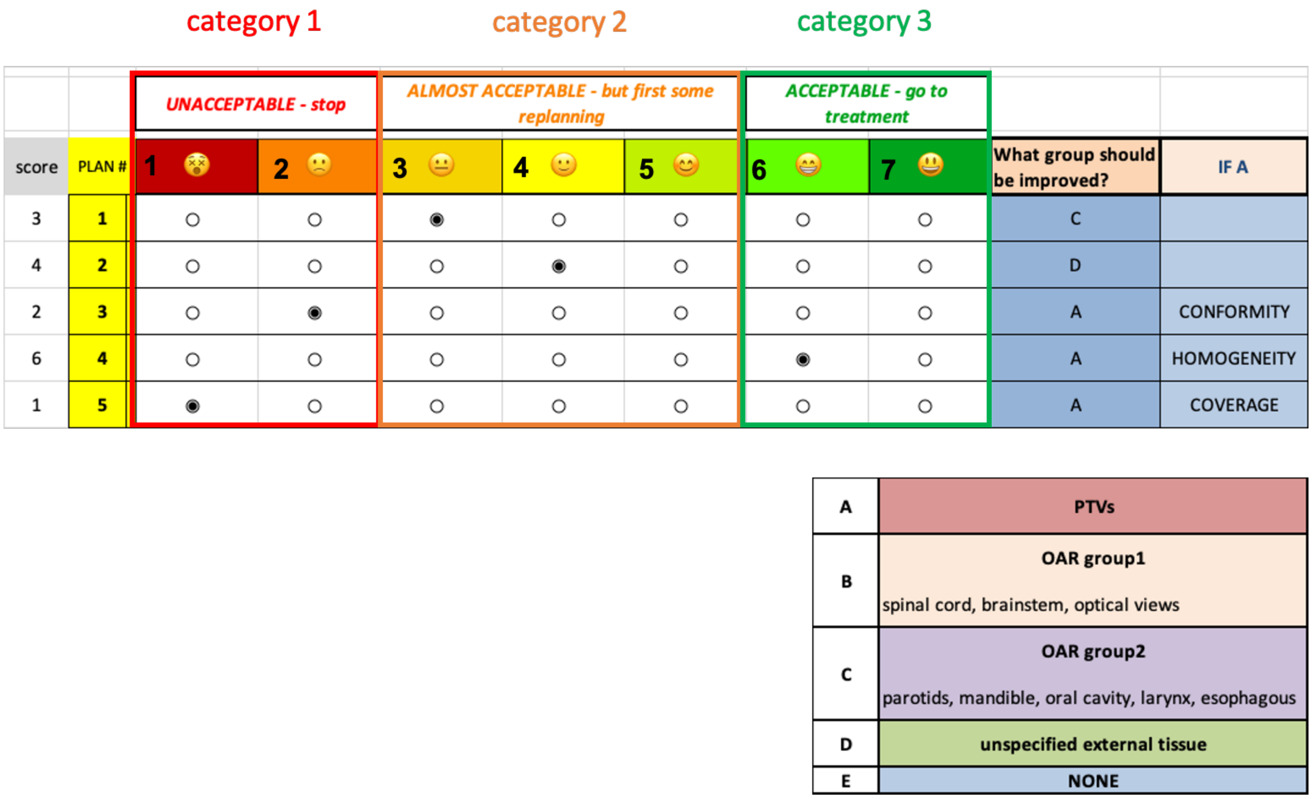

		category 1		category 2			category 3			
		UNACCEPTABLE - stop		ALMOST ACCEPTABLE - but first some replanning			ACCEPTABLE - go to treatment			
score	PLAN #	1	2	3	4	5	6	7	What group should be improved?	IF A
3	1	○	○	●	○	○	○	○	C	
4	2	○	○	○	●	○	○	○	D	
2	3	○	●	○	○	○	○	○	A	CONFORMITY
6	4	○	○	○	○	○	●	○	A	HOMOGENEITY
1	5	●	○	○	○	○	○	○	A	COVERAGE

A	PTVs
B	OAR group1<br>spinal cord, brainstem, optical views
C	OAR group2<br>parotids, mandible, oral cavity, larynx, esophagous
D	unspecified external tissue
E	NONE

Supplementary Figure S1. Sheet used for plan scoring and for indicating the most important suggestion for plan improvement. a) left part: filled-in scoring sheet of one of the observers for one of the study patients with 5 available plans. The same (empty) sheet was used by all observers for all patients. For each patient plan (first, yellow column), an observer had to choose a score between 1 and 7, with 1 and 7 lowest and highest quality, respectively. The scores were divided in three categories with explanations in the first row of the sheet. A score in category 2 meant that the plan would be acceptable if further planning would not result in desired improvements. right part: the observer also had to express the most desired plan improvement (without knowing whether it would be feasible or not). b) possible choices for plan improvements. PTVs (coverage, conformity, or homogeneity), OAR group1 (spinal cord, brainstem, optical system), OAR group 2 (parotids, mandible, oral cavity, larynx, esophagus), unspecified external tissue or NONE.

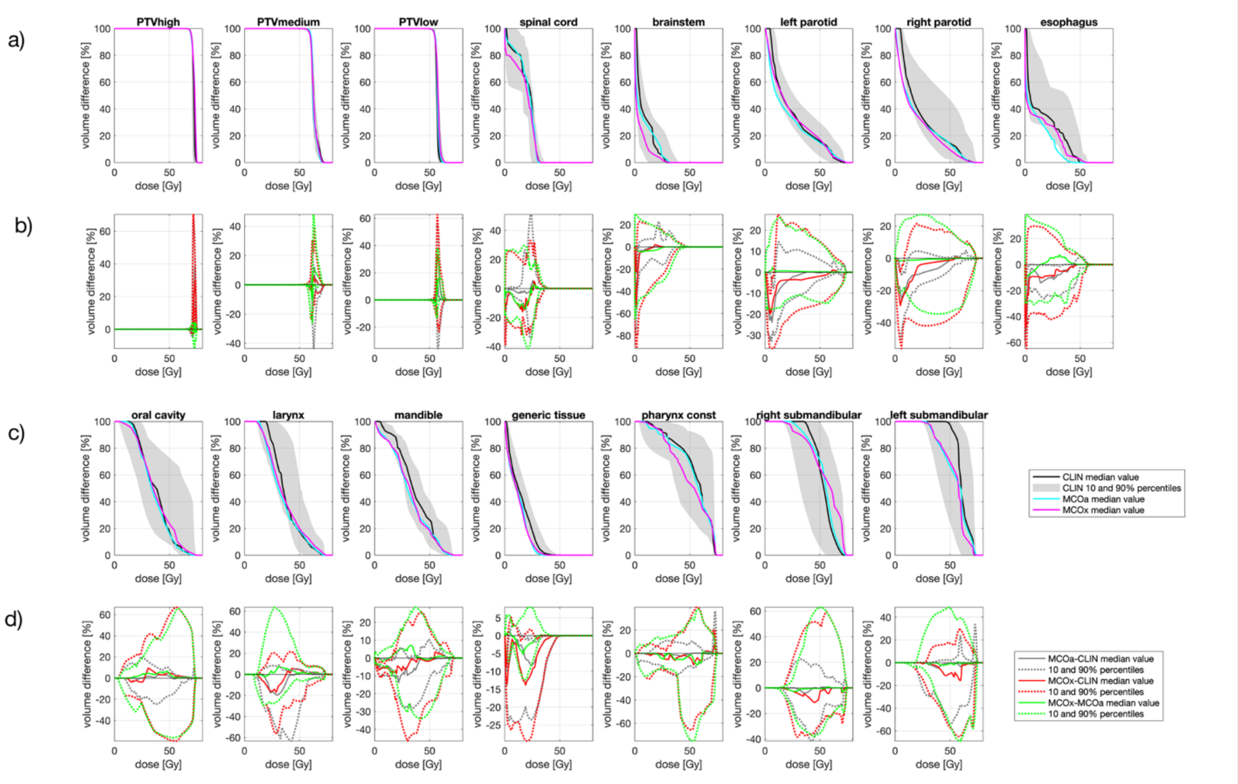

Supplementary Figure S2. a) and c): median DVHs for the 15 CLIN with 10-90% percentiles, 15 MCOa and 35 MCOx plans. b) and d): for each dose level (x-axis), median differences in DVH volumes with 10 and 90% percentiles.

Table S1. Median differences between the 65 included CLIN, MCOa and MCOx plans in dosimetric plan parameters with ranges and p-values. Significant p-values are reported in bold.

**a)**	**MCOa – CLIN**				**MCOx - CLIN**				**MCOx - MCOa**			
**endpoints**	**Diff (Gy)**	**min (Gy)**	**max (Gy)**	**p**	**Diff (Gy)**	**min (Gy)**	**max (Gy)**	**p**	**Diff (Gy)**	**min (Gy)**	**max (Gy)**	**p**
PTV1 D98%	-0.2	-0.9	0.7	0.2	-0.1	-0.8	0.5	0.3	0.2	-0.9	0.5	**0.02**
PTV1 Dmax	1.3	-11.4	7.6	0.1	1.2	-2.1	6.7	**<0.001**	0.0	-5.6	10.9	0.5
PTV2 D98%	0.6	-0.7	2.5	**0.02**	-0.1	-2.2	2.4	0.4	-0.7	-2.7	0.4	**<0.001**
PTV3 D98%	0.1	-1.5	2.0	0.8	0.1	-1.8	2.0	0.5	-0.2	-1.4	1.5	**0.02**
Spinal cord Dmax	-1.9	-6.0	9.7	0.2	0.0	-5.6	12.4	0.8	0.2	-3.3	9.1	0.2
Brainstem Dmax	1.0	-11.2	21.4	0.9	-1.8	-21.1	23.1	0.4	-0.4	-21.0	25.7	0.7
Left parotid Dmean	-5.0	-9.3	10.2	0.5	-3.7	-9.9	24.2	0.2	0.4	-4.9	27.2	0.5
Right parotid Dmean	-2.5	-8.1	7.1	0.6	-0.3	-8.2	21.9	0.9	0.2	-3.3	25.3	0.4
Esophagous Dmean	-4.0	-10.2	10.2	0.1	-1.6	-8.8	6.1	**0.02**	1.3	-9.7	10.1	0.1
Oral cavity Dmean	1.7	-6.3	9.4	0.6	2.5	-7.1	14.8	0.3	1.6	-9.3	7.7	0.7
Larynx Dmean	-3.2	-18.8	3.0	**0.003**	-1.9	-23.4	11.7	0.1	0.8	-14.4	16.8	0.1
Mandible Dmax	-1.3	-4.1	3.2	0.07	0.0	-2.6	2.8	0.9	1.9	-3.2	4.4	**0.004**
Generic tissue Dmean	-1.0	-7.3	8.4	**0.05**	-1.6	-9.3	0.2	**<0.001**	-0.3	-9.6	4.4	0.4
Pharynx constictors. Dmean	0.2	-9.4	4.7	0.9	0.4	-10.9	10.2	0.9	0.0	-10.0	9.8	0.9
Left submandibular Dmean	-1.9	-15.0	2.1	0.5	-4.2	-15.5	13.1	0.1	-0.5	-11.8	11.2	0.6
Right submandibular Dmean	-4.6	-17.1	1.1	0.2	-3.5	-16.3	3.5	0.1	-0.2	-15.9	14.1	0.9

Patient 1

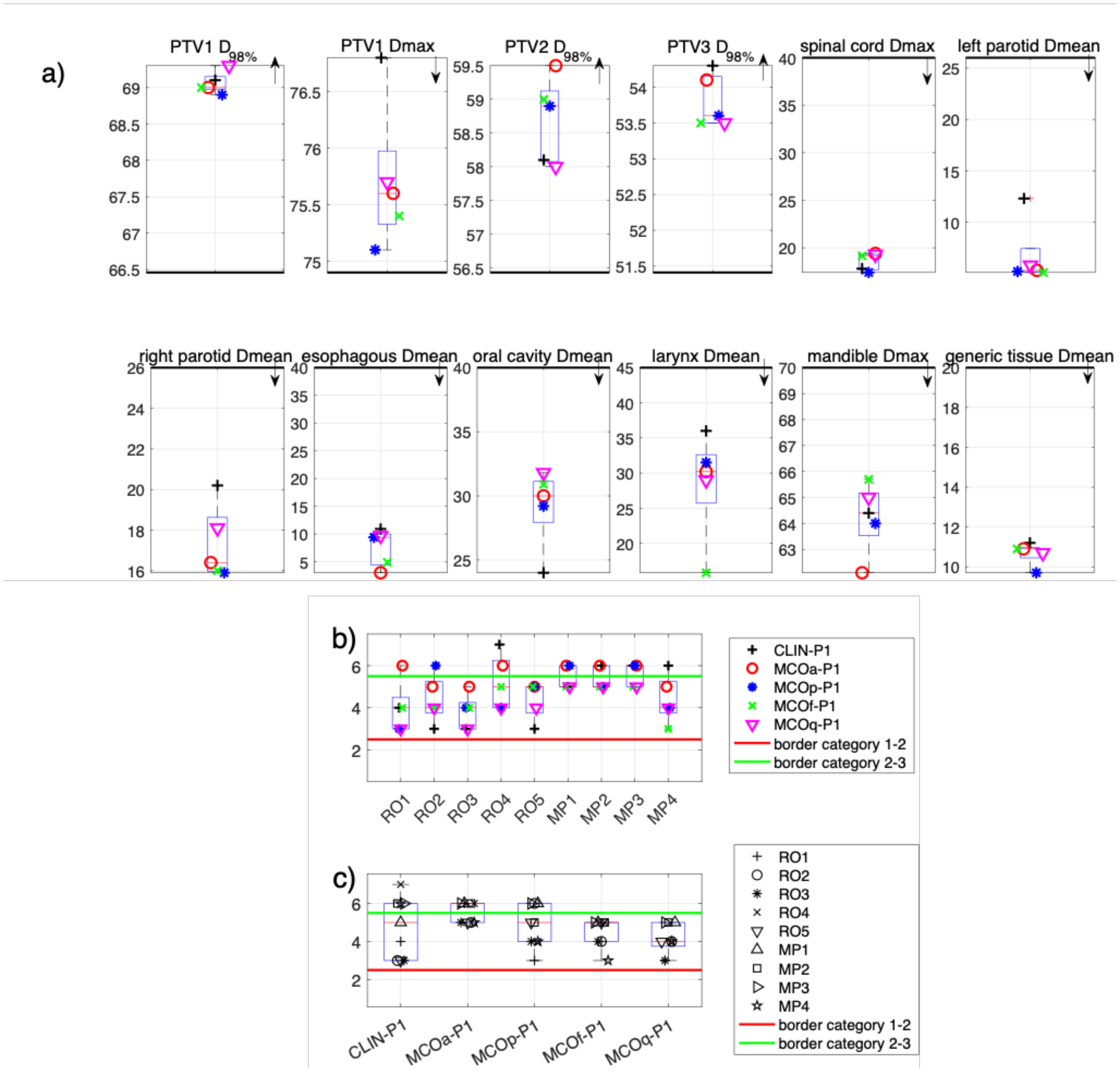
a)
PTV1 D98%
PTV1 Dmax
PTV2 D98%
PTV3 D98%
spinal cord Dmax
left parotid Dmean
right parotid Dmean
esophagous Dmean
oral cavity Dmean
larynx Dmean
mandible Dmax
generic tissue Dmean
b)
CLIN-P1
MCOa-P1
MCOp-P1
MCOf-P1
MCOq-P1
border category 1-2
border category 2-3
RO1
RO2
RO3
RO4
RO5
MP1
MP2
MP3
MP4
c)

Patient 2

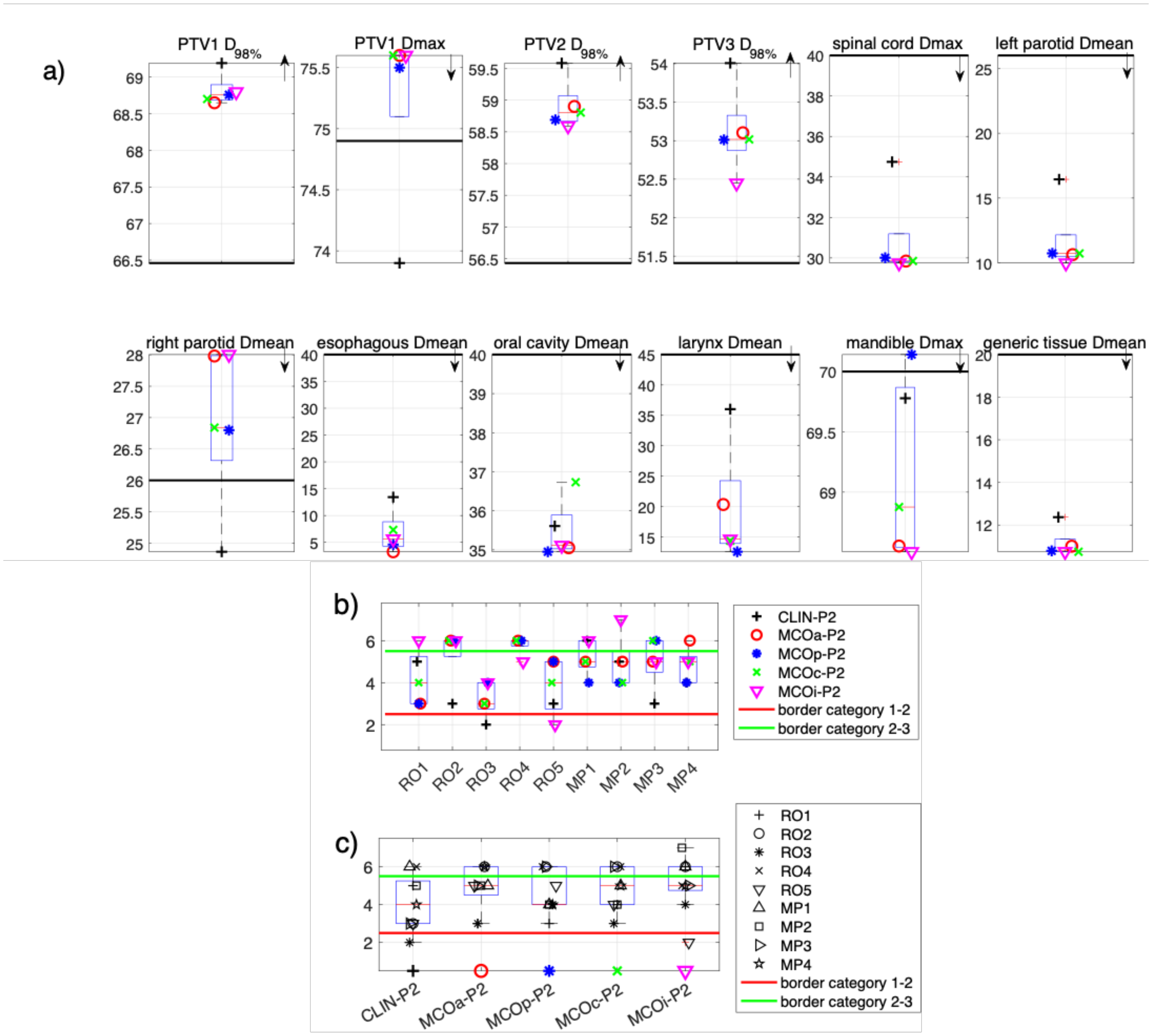
a)
PTV1 D98%
PTV1 Dmax
PTV2 D98%
PTV3 D98%
spinal cord Dmax
left parotid Dmean
right parotid Dmean
esophagous Dmean
oral cavity Dmean
larynx Dmean
mandible Dmax
generic tissue Dmean
b)
RO1
RO2
RO3
RO4
RO5
MP1
MP2
MP3
MP4
CLIN-P2
MCOa-P2
MCOp-P2
MCOc-P2
MCOi-P2
border category 1-2
border category 2-3
c)
CLIN-P2
MCOa-P2
MCOp-P2
MCOc-P2
MCOi-P2
RO1
RO2
RO3
RO4
RO5
MP1
MP2
MP3
MP4
border category 1-2
border category 2-3

Patient 3

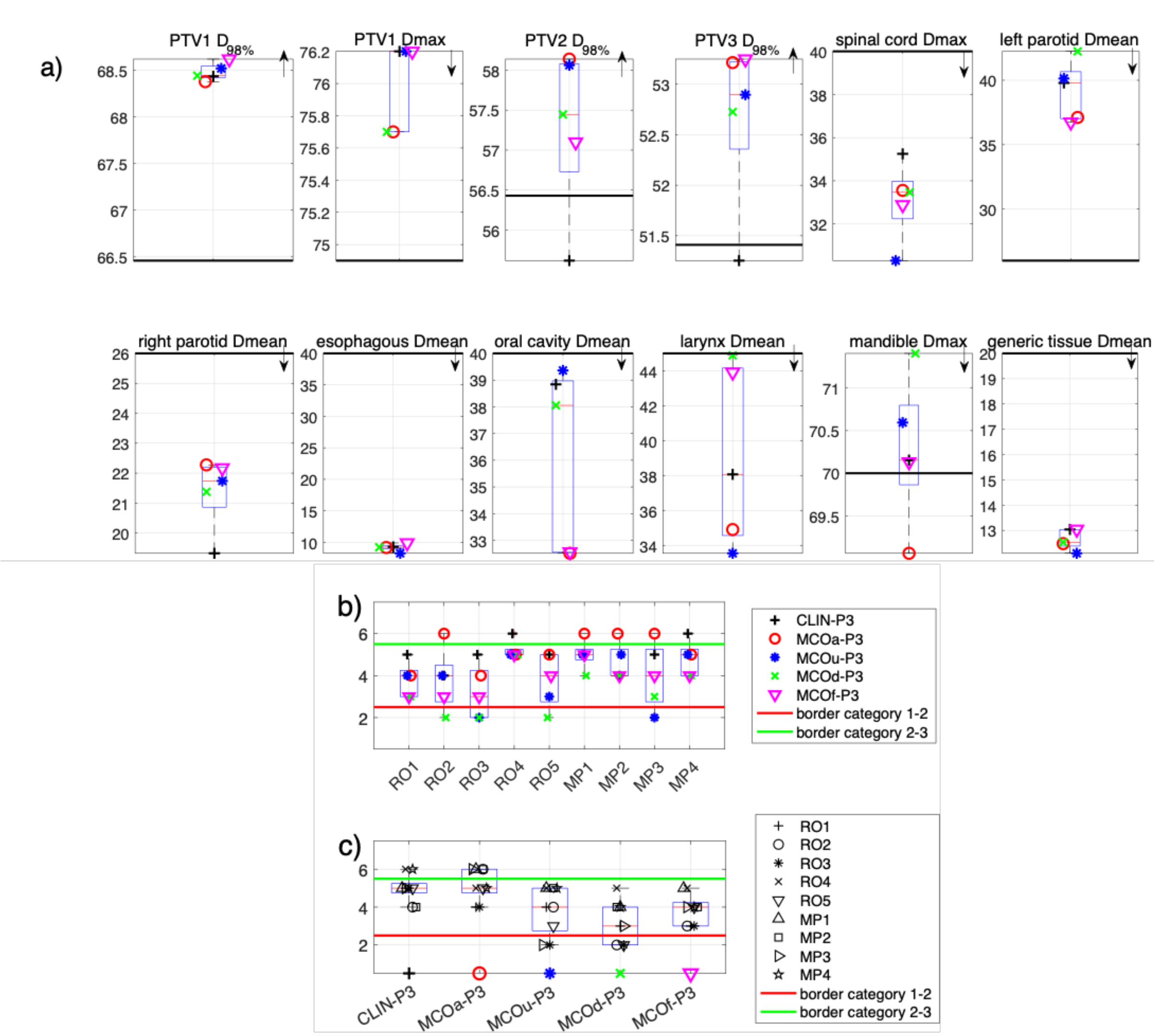
a)
PTV1 D98%
PTV1 Dmax
PTV2 D98%
PTV3 D98%
spinal cord Dmax
left parotid Dmean
right parotid Dmean
esophagous Dmean
oral cavity Dmean
larynx Dmean
mandible Dmax
generic tissue Dmean
b)
CLIN-P3
MCOa-P3
MCOu-P3
MCOd-P3
MCOf-P3
border category 1-2
border category 2-3
RO1 RO2 RO3 RO4 RO5 MP1 MP2 MP3 MP4
c)
RO1
RO2
RO3
RO4
RO5
MP1
MP2
MP3
MP4
border category 1-2
border category 2-3
CLIN-P3 MCOa-P3 MCOu-P3 MCOd-P3 MCOf-P3

Patient 4

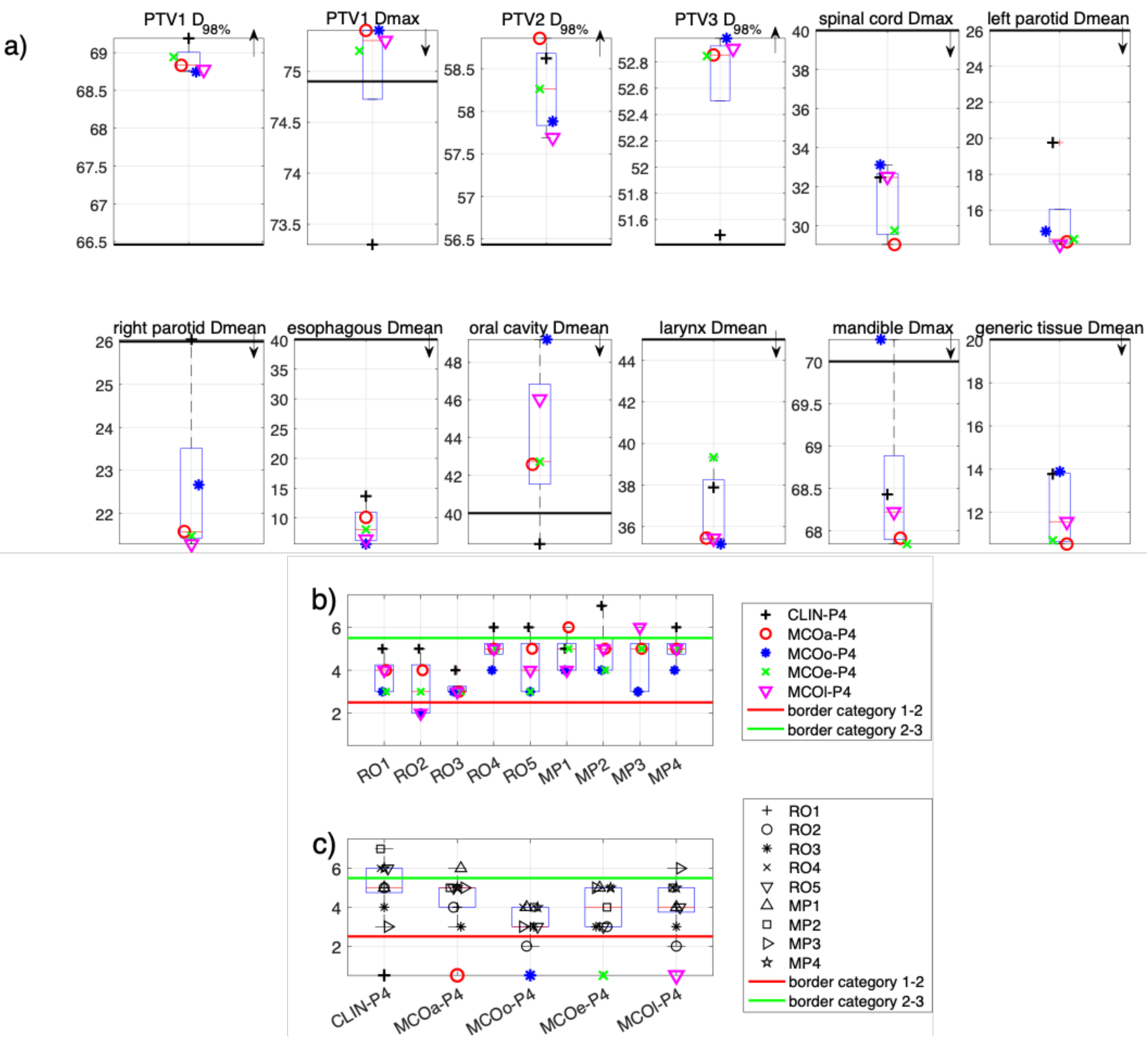

a)
PTV1 D98%
PTV1 Dmax
PTV2 D98%
PTV3 D98%
spinal cord Dmax
left parotid Dmean
right parotid Dmean
esophagous Dmean
oral cavity Dmean
larynx Dmean
mandible Dmax
generic tissue Dmean
b)
RO1
RO2
RO3
RO4
RO5
MP1
MP2
MP3
MP4
CLIN-P4
MCOa-P4
MCOo-P4
MCOe-P4
MCOl-P4
border category 1-2
border category 2-3
c)
CLIN-P4
MCOa-P4
MCOo-P4
MCOe-P4
MCOl-P4
RO1
RO2
RO3
RO4
RO5
MP1
MP2
MP3
MP4
border category 1-2
border category 2-3

Patient 5

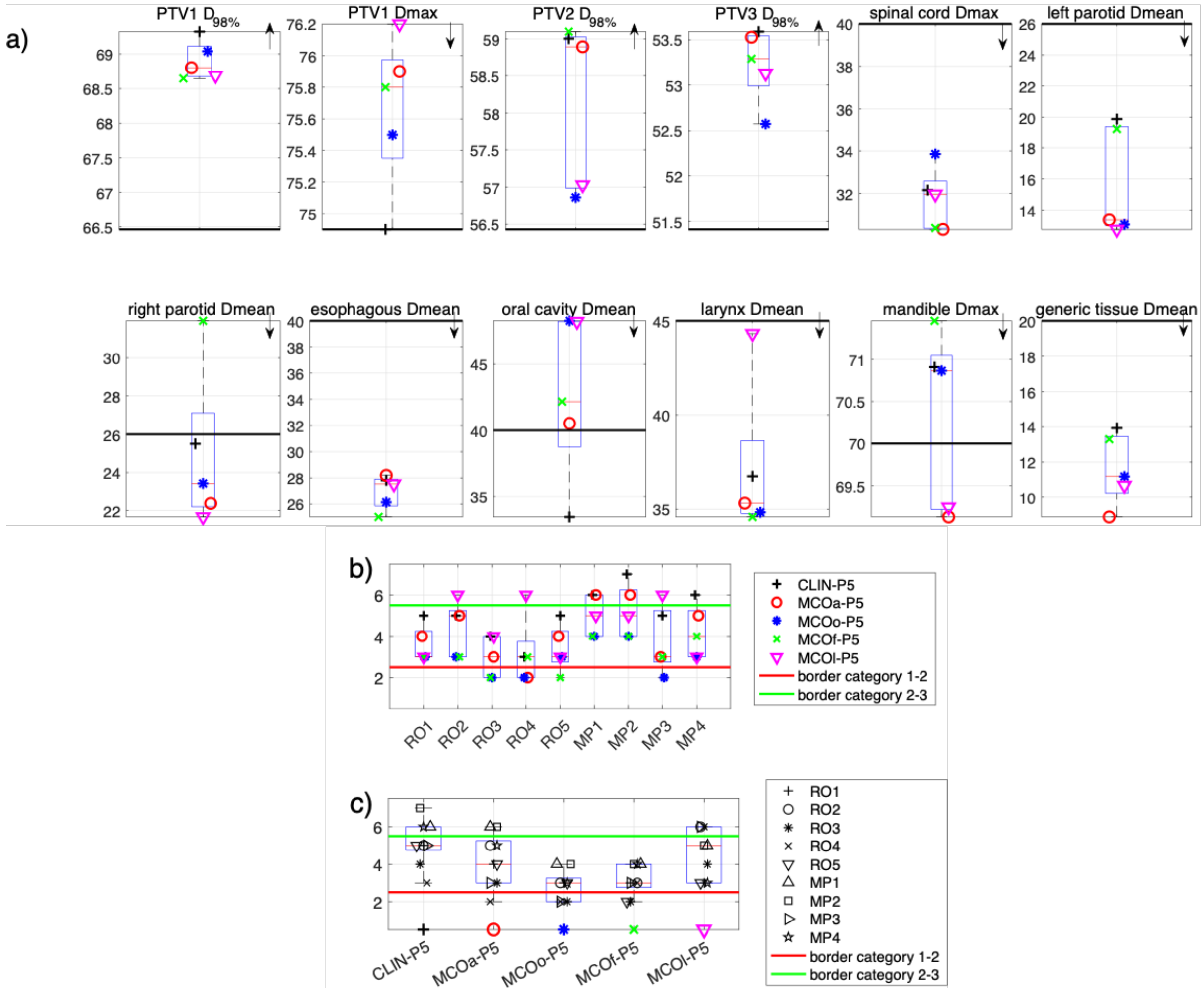
a)
PTV1 D98%
PTV1 Dmax
PTV2 D98%
PTV3 D98%
spinal cord Dmax
left parotid Dmean
right parotid Dmean
esophagous Dmean
oral cavity Dmean
larynx Dmean
mandible Dmax
generic tissue Dmean
b)
CLIN-P5
MCOa-P5
MCOo-P5
MCOf-P5
MCOl-P5
border category 1-2
border category 2-3
RO1
RO2
RO3
RO4
RO5
MP1
MP2
MP3
MP4
c)
CLIN-P5
MCOa-P5
MCOo-P5
MCOf-P5
MCOl-P5

Patient 6

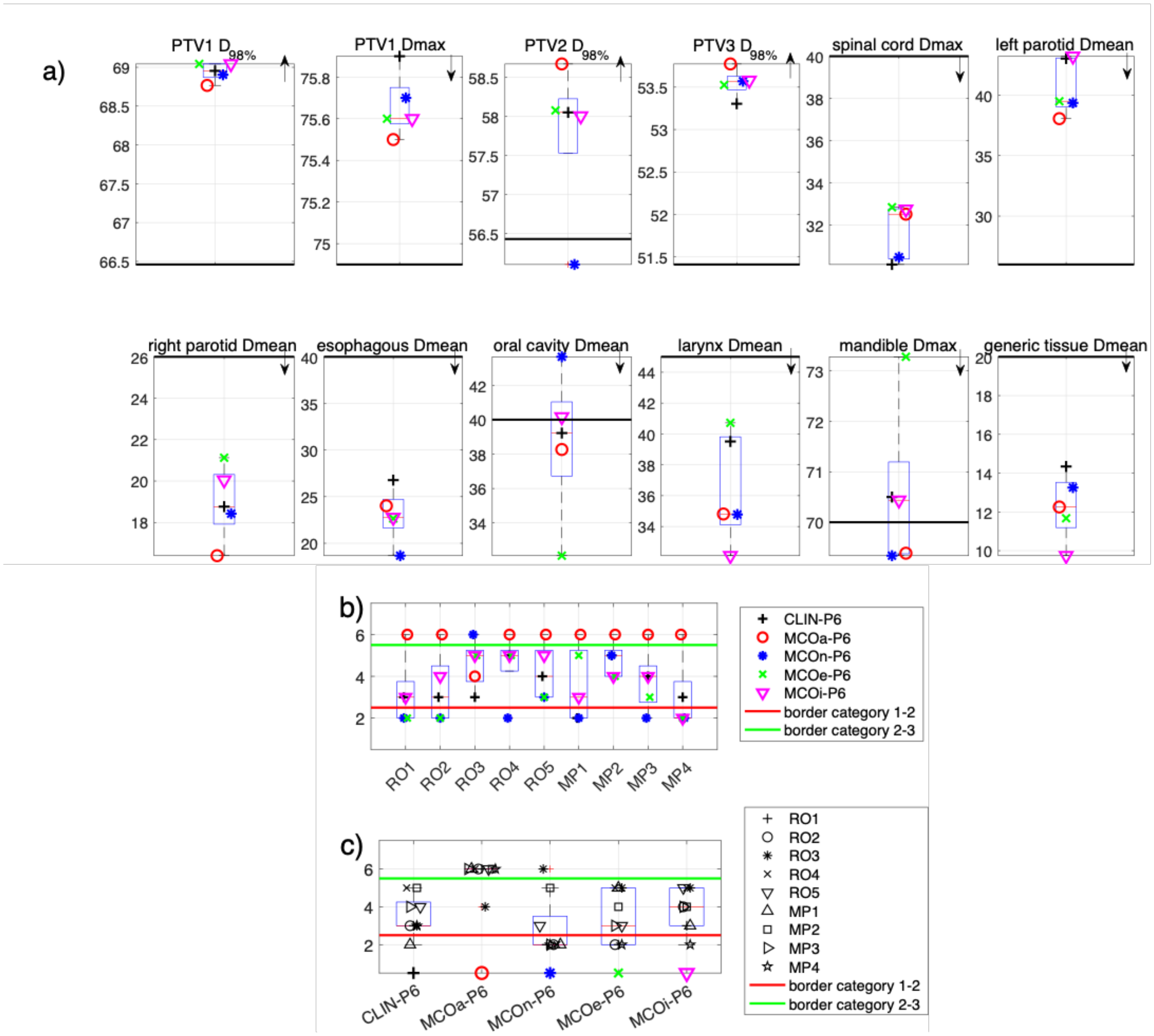

a)
PTV1 D98%
PTV1 Dmax
PTV2 D98%
PTV3 D98%
spinal cord Dmax
left parotid Dmean
right parotid Dmean
esophagous Dmean
oral cavity Dmean
larynx Dmean
mandible Dmax
generic tissue Dmean
b)
RO1
RO2
RO3
RO4
RO5
MP1
MP2
MP3
MP4
CLIN-P6
MCOa-P6
MCOn-P6
MCOe-P6
MCOi-P6
border category 1-2
border category 2-3
c)
CLIN-P6
MCOa-P6
MCOn-P6
MCOe-P6
MCOi-P6
RO1
RO2
RO3
RO4
RO5
MP1
MP2
MP3
MP4
border category 1-2
border category 2-3

Patient 7

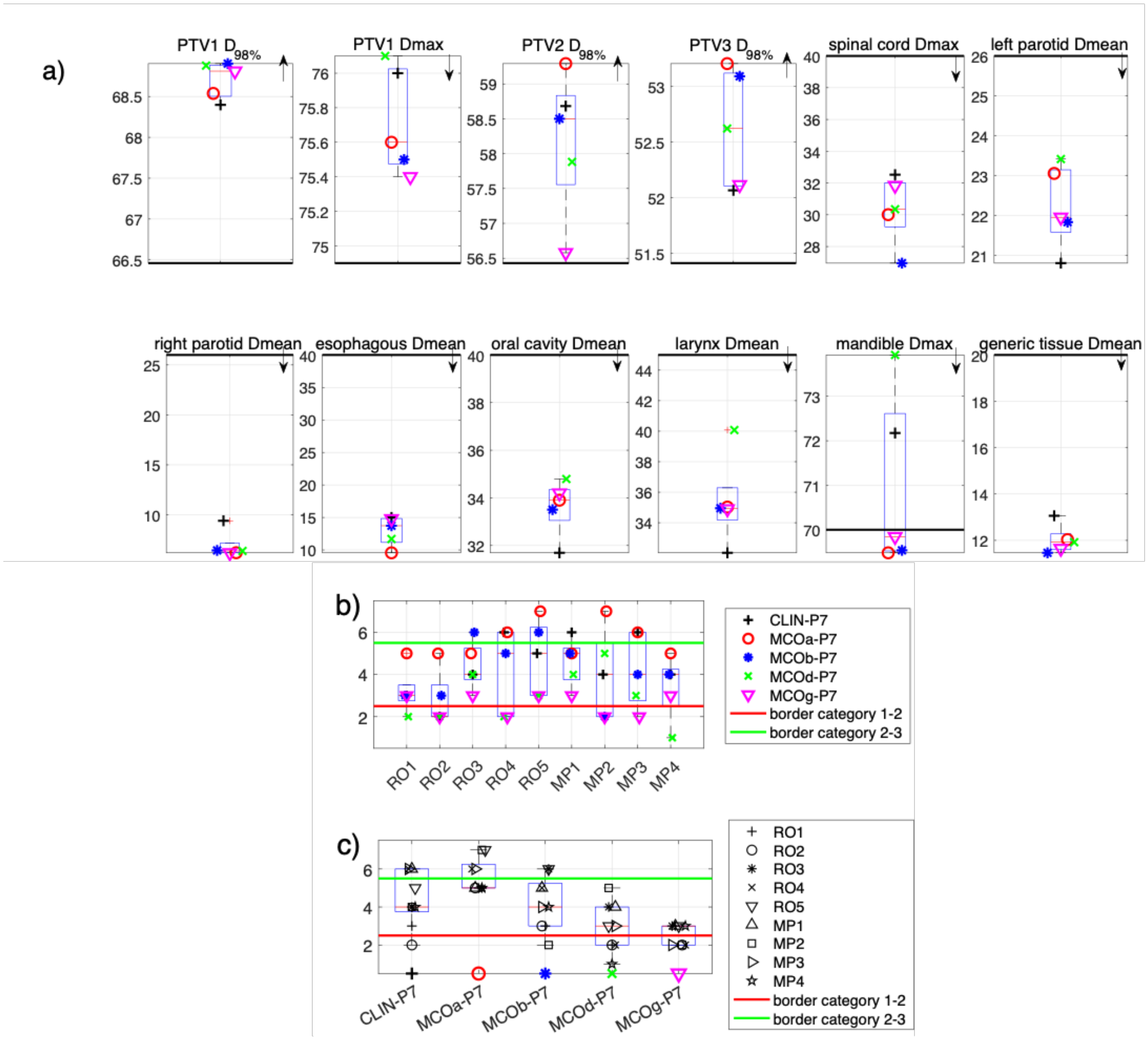
a)
PTV1 D98%
PTV1 Dmax
PTV2 D98%
PTV3 D98%
spinal cord Dmax
left parotid Dmean
right parotid Dmean
esophagous Dmean
oral cavity Dmean
larynx Dmean
mandible Dmax
generic tissue Dmean
b)
RO1
RO2
RO3
RO4
RO5
MP1
MP2
MP3
MP4
CLIN-P7
MCOa-P7
MCOb-P7
MCOd-P7
MCOg-P7
border category 1-2
border category 2-3
c)
RO1
RO2
RO3
RO4
RO5
MP1
MP2
MP3
MP4
border category 1-2
border category 2-3

Patient 8

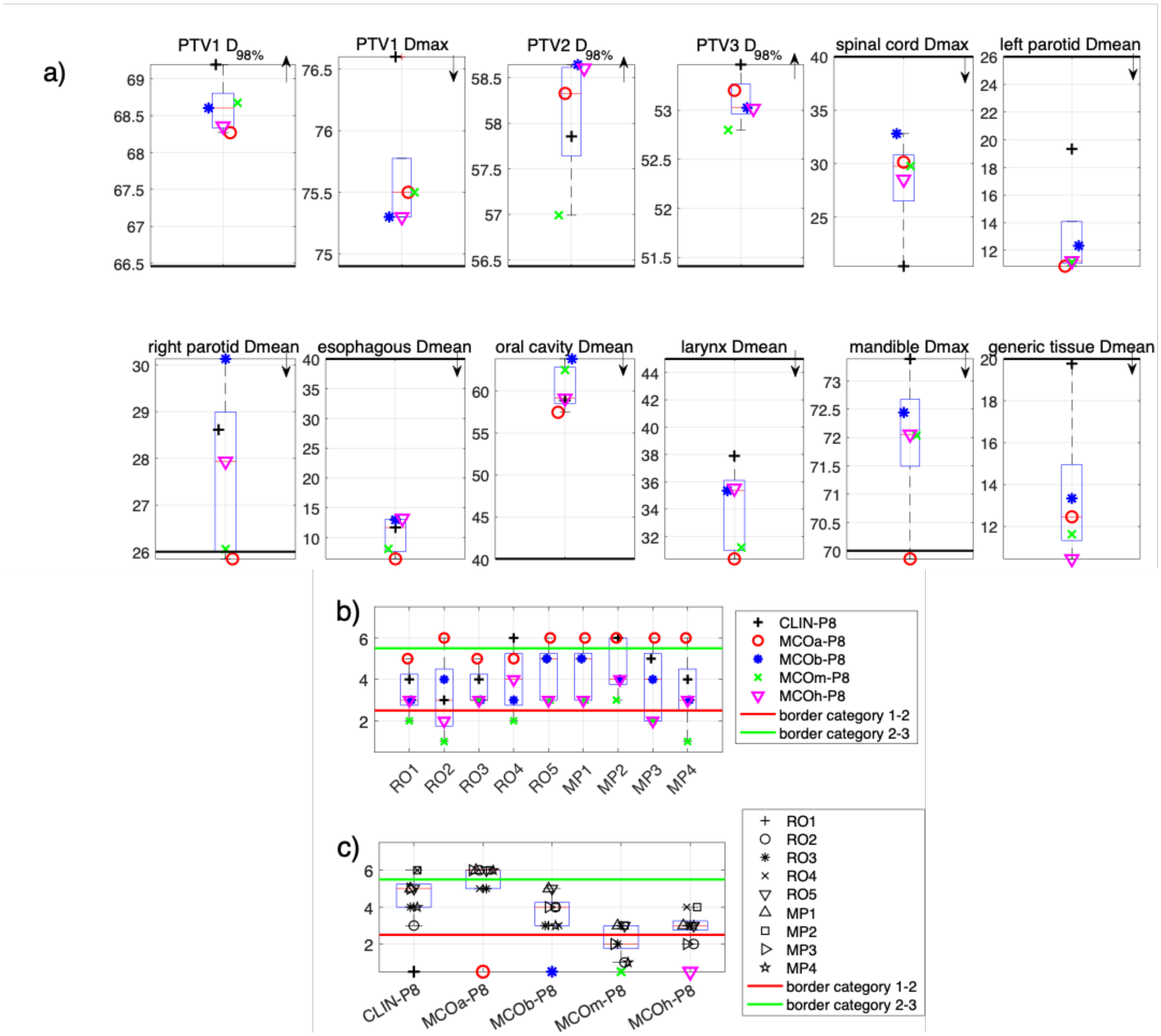

a)
PTV1 D98%
PTV1 Dmax
PTV2 D98%
PTV3 D98%
spinal cord Dmax
left parotid Dmean
right parotid Dmean
esophagous Dmean
oral cavity Dmean
larynx Dmean
mandible Dmax
generic tissue Dmean
b)
CLIN-P8
MCOa-P8
MCOb-P8
MCOm-P8
MCOh-P8
border category 1-2
border category 2-3
RO1
RO2
RO3
RO4
RO5
MP1
MP2
MP3
MP4
c)
CLIN-P8
MCOa-P8
MCOb-P8
MCOm-P8
MCOh-P8
RO1
RO2
RO3
RO4
RO5
MP1
MP2
MP3
MP4
border category 1-2
border category 2-3

Patient 9

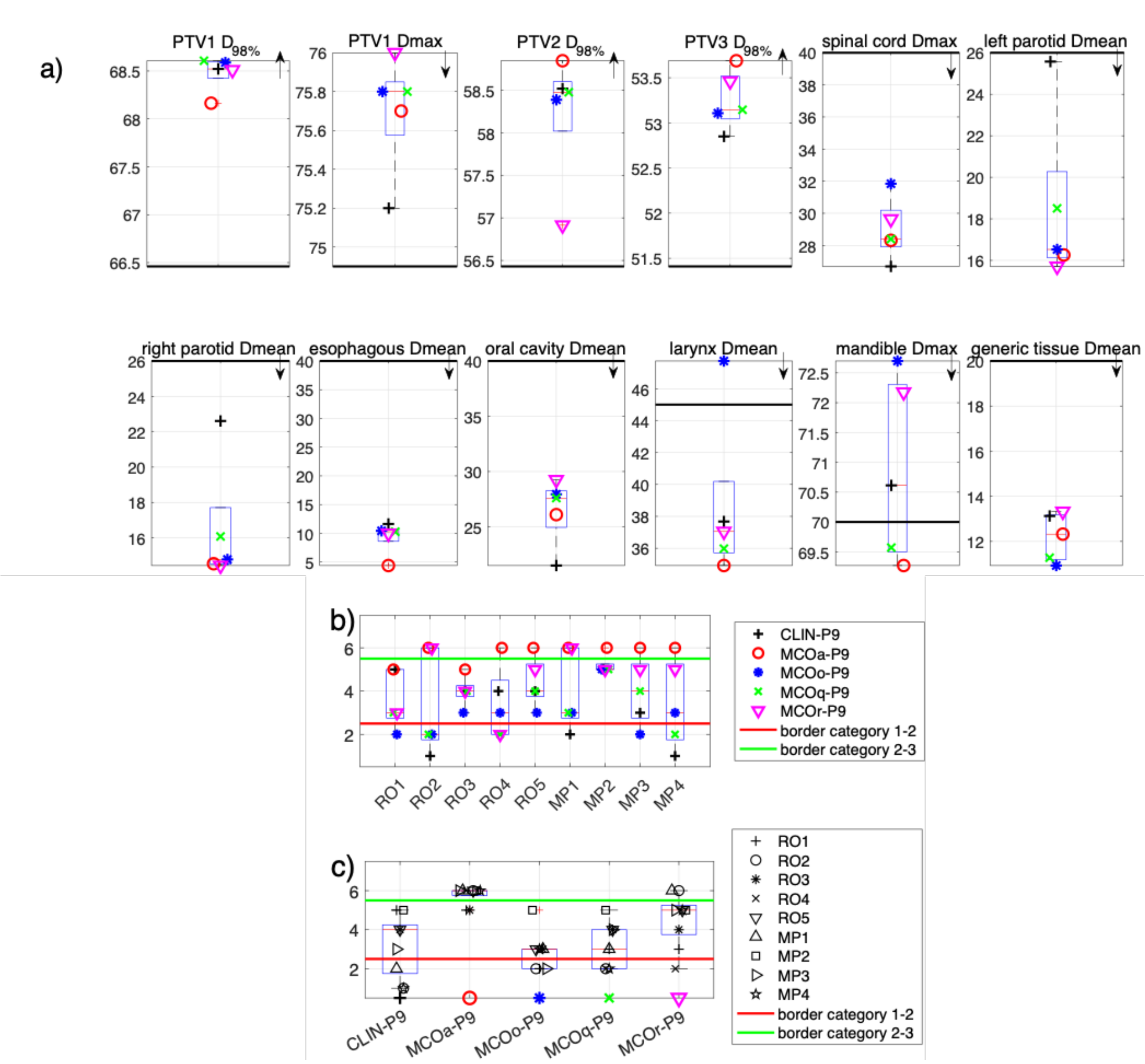
a)
PTV1 D98%
PTV1 Dmax
PTV2 D98%
PTV3 D98%
spinal cord Dmax
left parotid Dmean
right parotid Dmean
esophagous Dmean
oral cavity Dmean
larynx Dmean
mandible Dmax
generic tissue Dmean
b)
RO1
RO2
RO3
RO4
RO5
MP1
MP2
MP3
MP4
CLIN-P9
MCOa-P9
MCOo-P9
MCOq-P9
MCOr-P9
border category 1-2
border category 2-3
c)
CLIN-P9
MCOa-P9
MCOo-P9
MCOq-P9
MCOr-P9
RO1
RO2
RO3
RO4
RO5
MP1
MP2
MP3
MP4
border category 1-2
border category 2-3

Patient 10

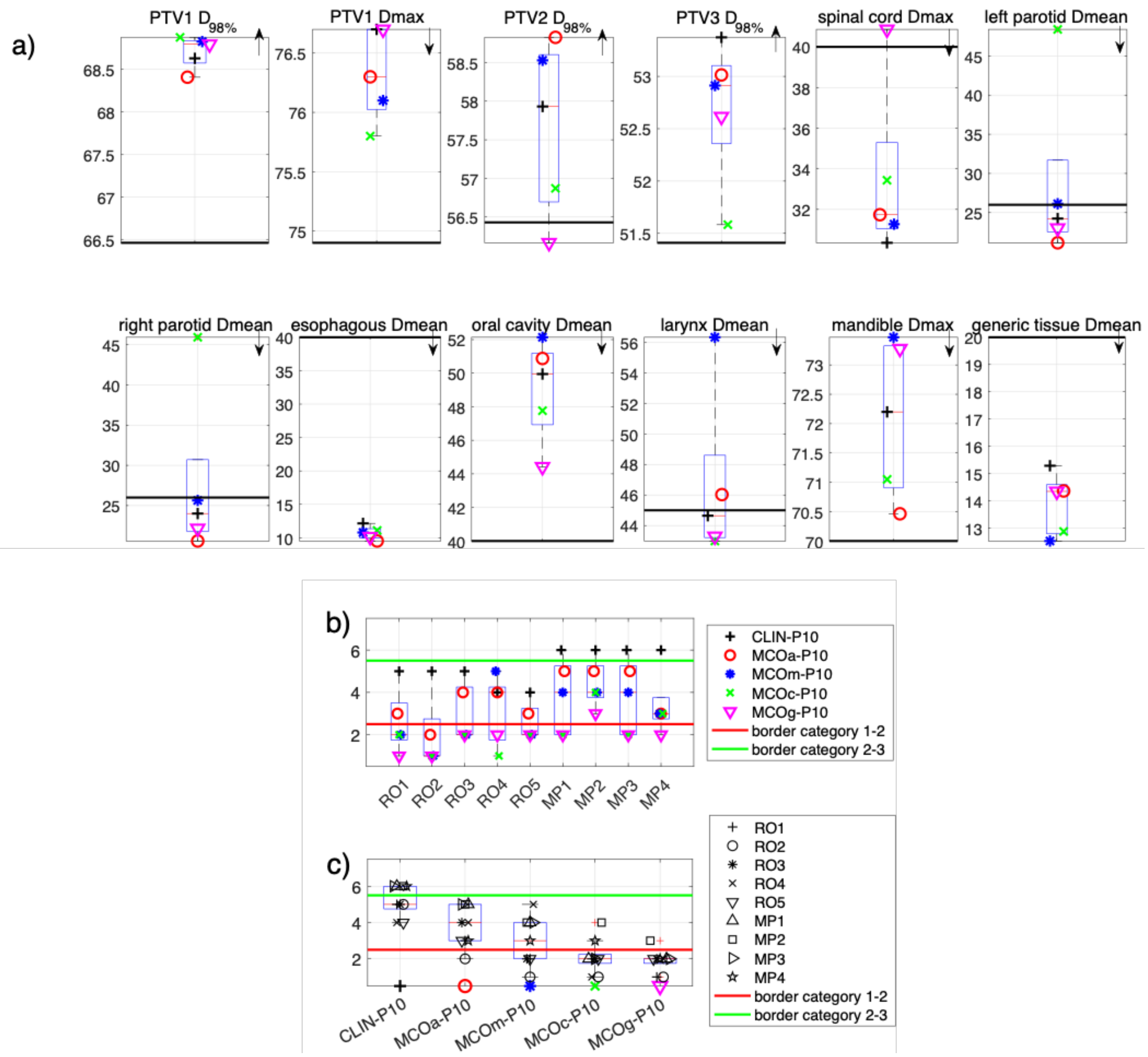
a)
PTV1 $D_{98\%}$
PTV1 Dmax
PTV2 $D_{98\%}$
PTV3 $D_{98\%}$
spinal cord Dmax
left parotid Dmean
right parotid Dmean
esophagous Dmean
oral cavity Dmean
larynx Dmean
mandible Dmax
generic tissue Dmean
b)
CLIN-P10
MCOa-P10
MCOm-P10
MCOc-P10
MCOg-P10
border category 1-2
border category 2-3
RO1
RO2
RO3
RO4
RO5
MP1
MP2
MP3
MP4
c)
RO1
RO2
RO3
RO4
RO5
MP1
MP2
MP3
MP4
border category 1-2
border category 2-3
CLIN-P10
MCOa-P10
MCOm-P10
MCOc-P10
MCOg-P10

Patient 11

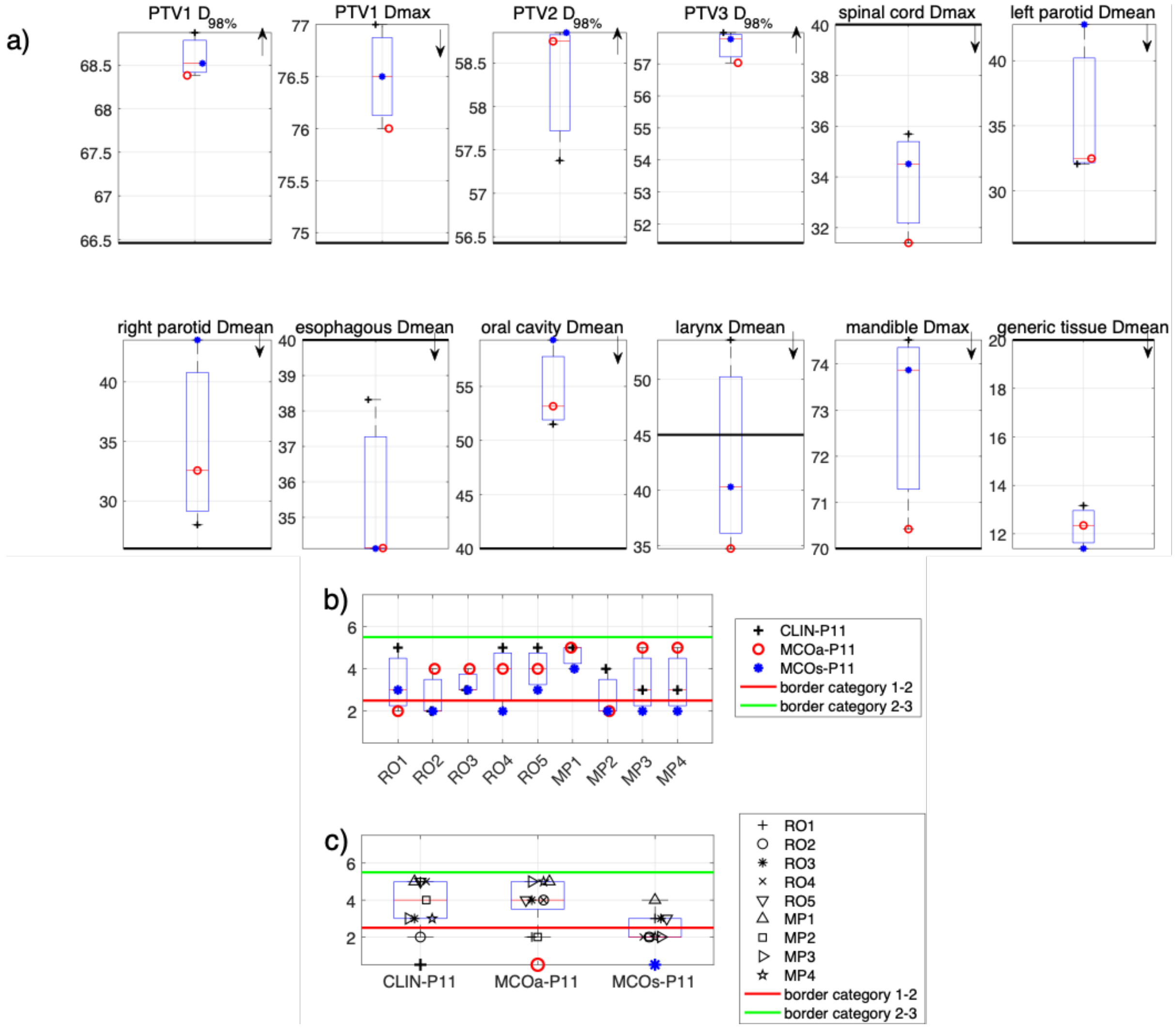
a)
PTV1 D98%
PTV1 Dmax
PTV2 D98%
PTV3 D98%
spinal cord Dmax
left parotid Dmean
right parotid Dmean
esophagous Dmean
oral cavity Dmean
larynx Dmean
mandible Dmax
generic tissue Dmean
b)
RO1
RO2
RO3
RO4
RO5
MP1
MP2
MP3
MP4
CLIN-P11
MCOa-P11
MCOs-P11
border category 1-2
border category 2-3
c)
CLIN-P11
MCOa-P11
MCOs-P11
RO1
RO2
RO3
RO4
RO5
MP1
MP2
MP3
MP4
border category 1-2
border category 2-3

Patient 12

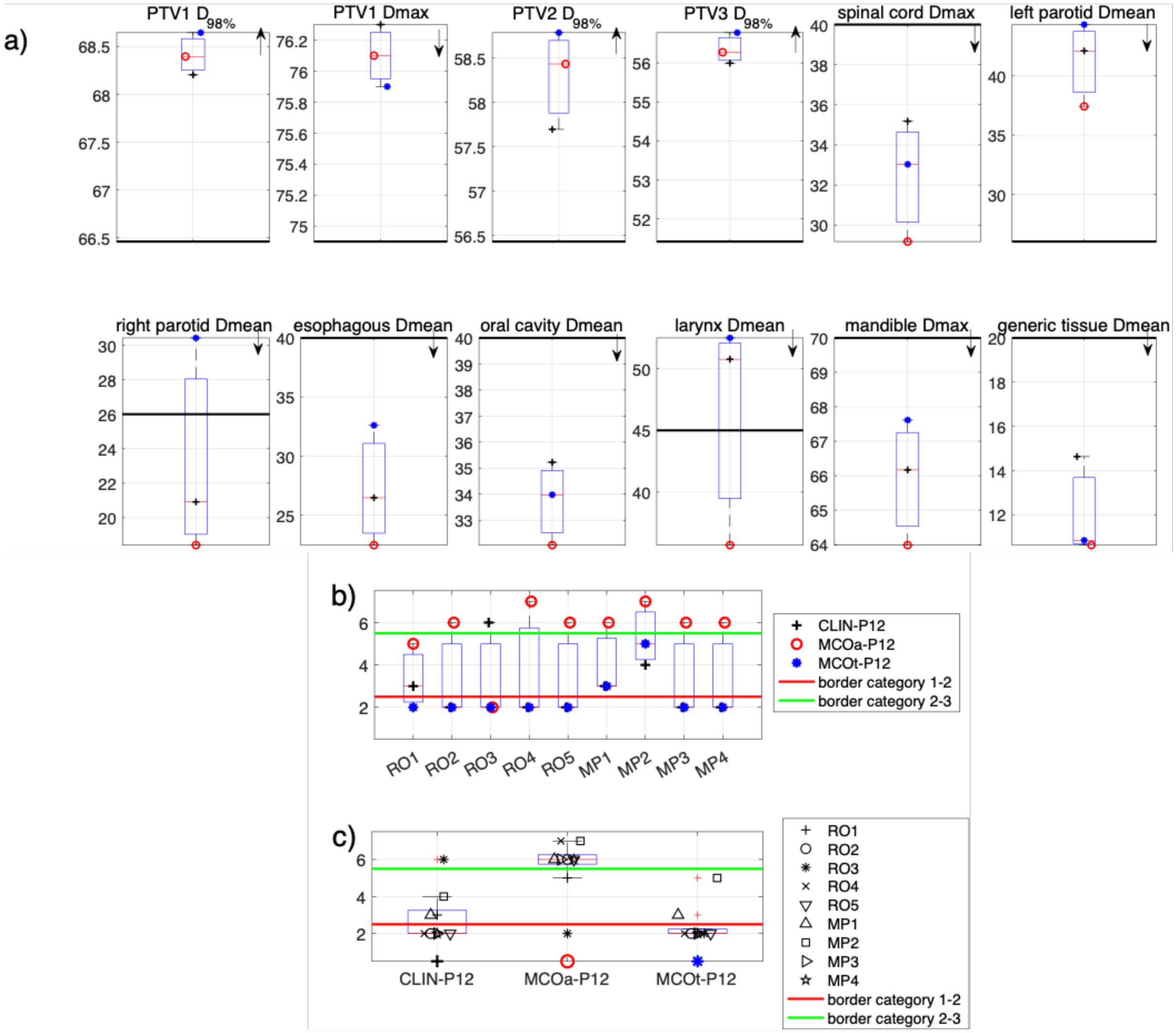

a)
PTV1 D98%
PTV1 Dmax
PTV2 D98%
PTV3 D98%
spinal cord Dmax
left parotid Dmean
right parotid Dmean
esophagous Dmean
oral cavity Dmean
larynx Dmean
mandible Dmax
generic tissue Dmean
b)
CLIN-P12
MCOa-P12
MCOt-P12
border category 1-2
border category 2-3
RO1
RO2
RO3
RO4
RO5
MP1
MP2
MP3
MP4
c)
RO1
RO2
RO3
RO4
RO5
MP1
MP2
MP3
MP4
border category 1-2
border category 2-3
CLIN-P12
MCOa-P12
MCOt-P12

Patient 13

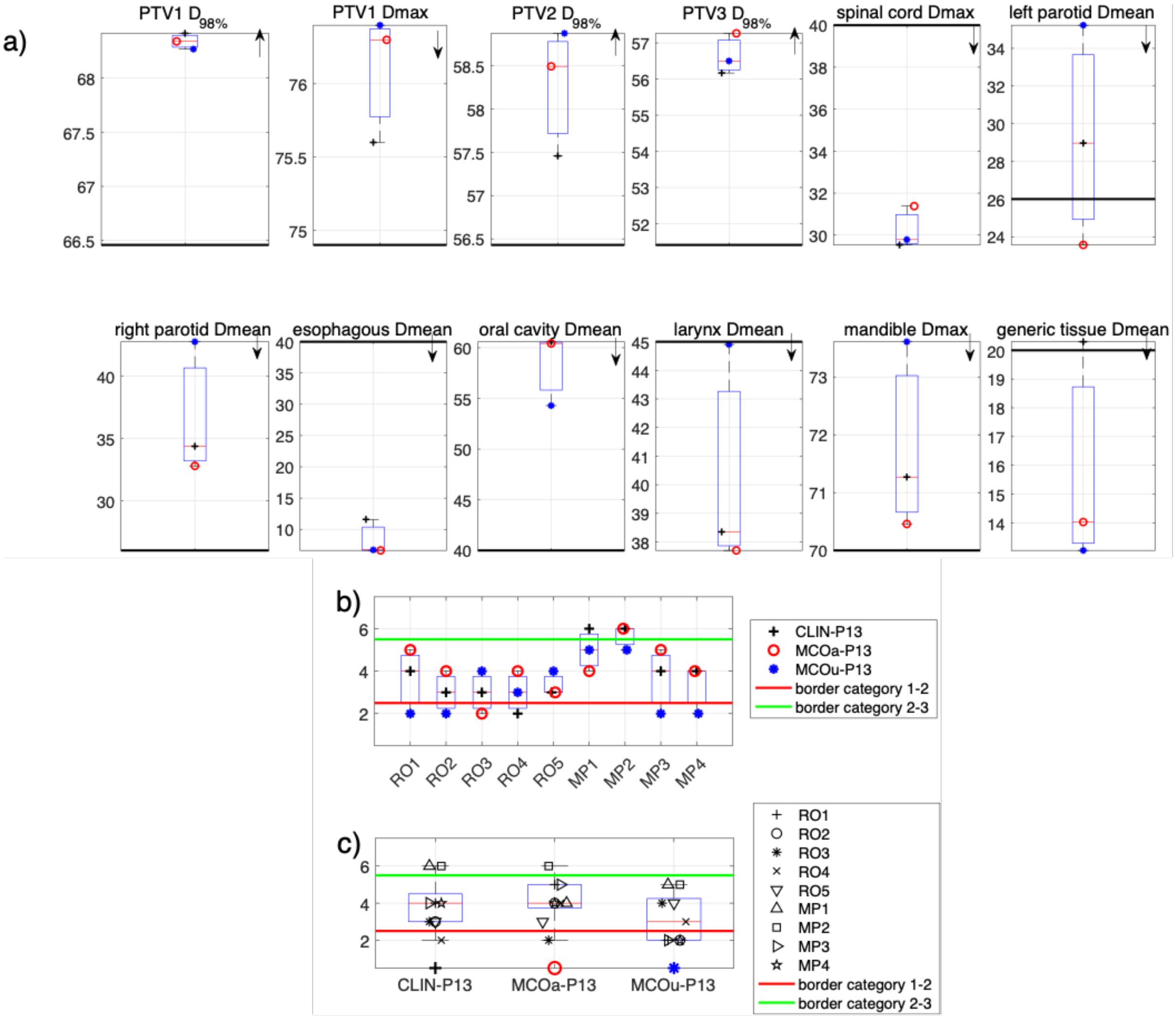
a)
PTV1 D98%
PTV1 Dmax
PTV2 D98%
PTV3 D98%
spinal cord Dmax
left parotid Dmean
right parotid Dmean
esophagous Dmean
oral cavity Dmean
larynx Dmean
mandible Dmax
generic tissue Dmean
b)
RO1 RO2 RO3 RO4 RO5 MP1 MP2 MP3 MP4
CLIN-P13
MCOa-P13
MCOu-P13
border category 1-2
border category 2-3
c)
CLIN-P13 MCOa-P13 MCOu-P13
RO1
RO2
RO3
RO4
RO5
MP1
MP2
MP3
MP4
border category 1-2
border category 2-3

Patient 14

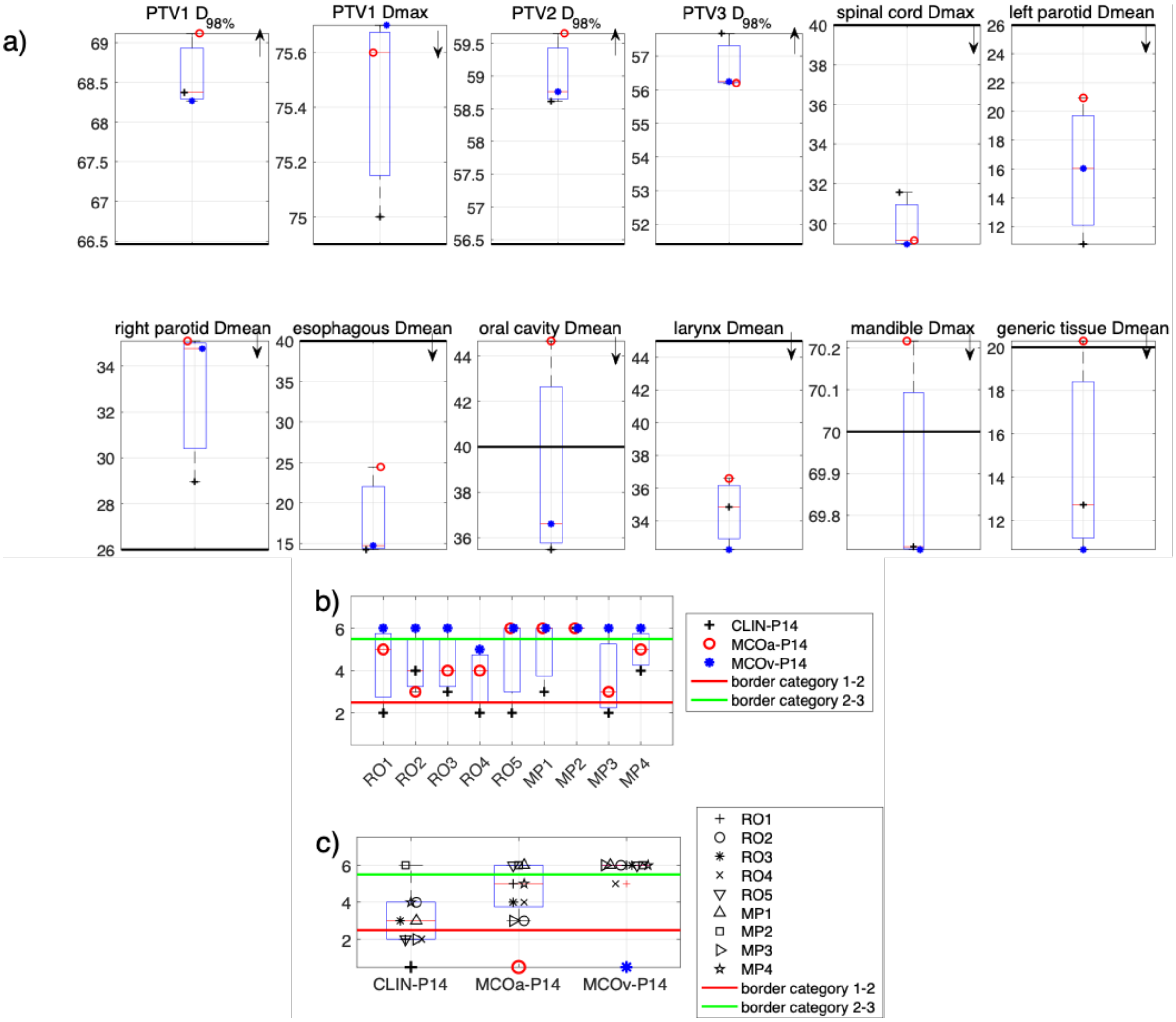

a)
PTV1 D98%
PTV1 Dmax
PTV2 D98%
PTV3 D98%
spinal cord Dmax
left parotid Dmean
right parotid Dmean
esophagous Dmean
oral cavity Dmean
larynx Dmean
mandible Dmax
generic tissue Dmean
b)
CLIN-P14
MCOa-P14
MCOv-P14
border category 1-2
border category 2-3
RO1 RO2 RO3 RO4 RO5 MP1 MP2 MP3 MP4
c)
RO1
RO2
RO3
RO4
RO5
MP1
MP2
MP3
MP4
border category 1-2
border category 2-3
CLIN-P14 MCOa-P14 MCOv-P14

Patient 15

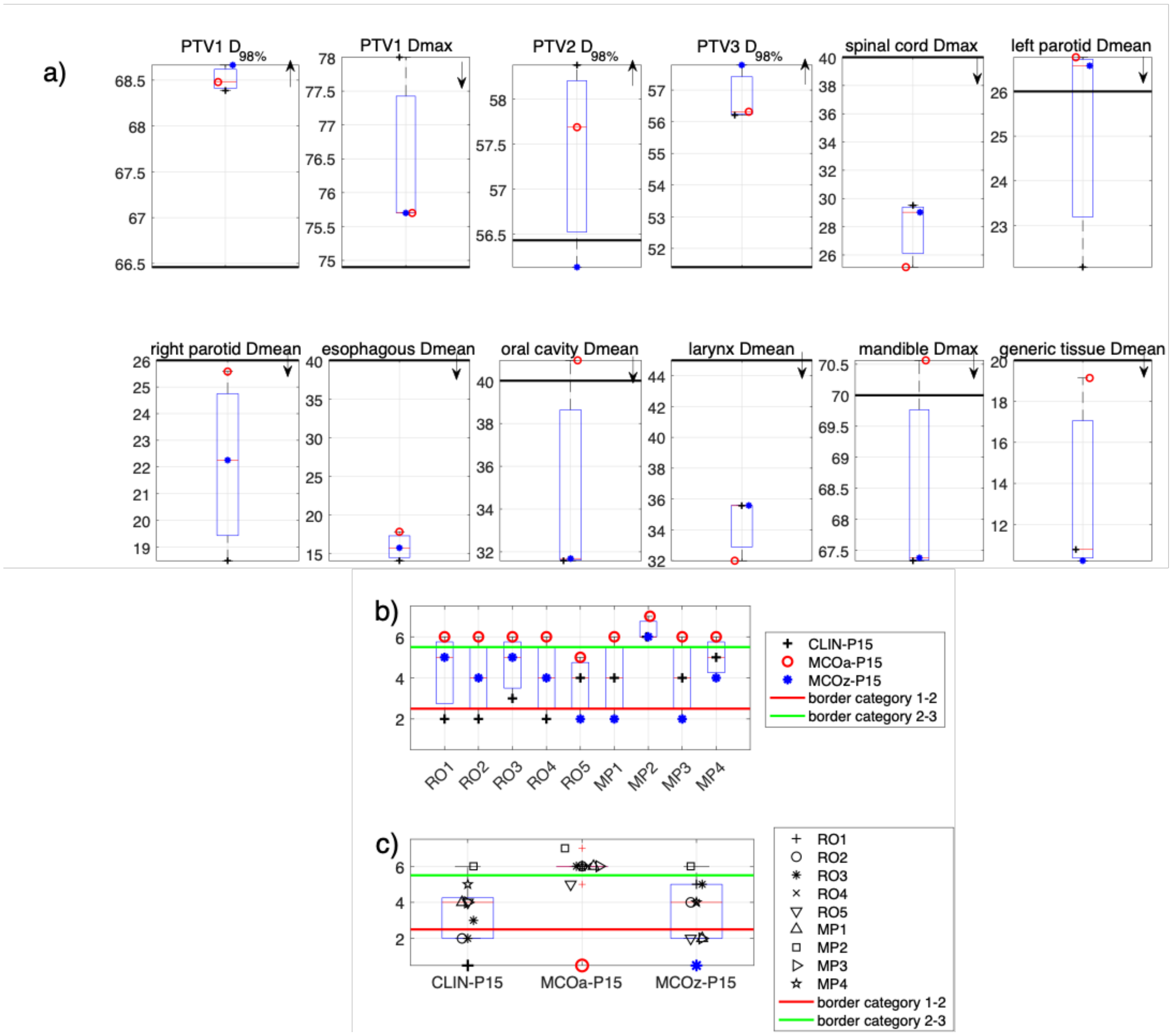

Supplementary Figure S3. For each of the 15 study patients, dosimetric parameters (a)) and subjective scores (b) and c)) of the 5 or 3 available treatment plans. In a), arrows indicate where plans should be relative to the black horizontal constraint lines. b) shows for each observer the scores for all available plans, while c) shows for each available plan the scores by all observers.
